# Genetic Circuits that Govern Bisexual and Unisexual Reproduction in *Cryptococcus neoformans*

**DOI:** 10.1371/journal.pgen.1003688

**Published:** 2013-08-15

**Authors:** Marianna Feretzaki, Joseph Heitman

**Affiliations:** 1 Department of Molecular Genetics and Microbiology, Duke University Medical Center, Durham, North Carolina, United States of America; 2 Department of Medicine, Duke University Medical Center, Durham, North Carolina, United States of America; 3 Department of Pharmacology and Cancer Biology, Duke University Medical Center, Durham, North Carolina, United States of America; Washington University, United States of America

## Abstract

*Cryptococcus neoformans* is a human fungal pathogen with a defined sexual cycle. Nutrient-limiting conditions and pheromones induce a dimorphic transition from unicellular yeast to multicellular hyphae and the production of infectious spores. Sexual reproduction involves cells of either opposite (bisexual) or one (unisexual) mating type. Bisexual and unisexual reproduction are governed by shared components of the conserved pheromone-sensing Cpk1 MAPK signal transduction cascade and by Mat2, the major transcriptional regulator of the pathway. However, the downstream targets of the pathway are largely unknown, and homology-based approaches have failed to yield downstream transcriptional regulators or other targets. In this study, we applied insertional mutagenesis via *Agrobacterium tumefaciens* transkingdom DNA delivery to identify mutants with unisexual reproduction defects. In addition to elements known to be involved in sexual development (Crg1, Ste7, Mat2, and Znf2), three key regulators of sexual development were identified by our screen: Znf3, Spo11, and Ubc5. Spo11 and Ubc5 promote sporulation during both bisexual and unisexual reproduction. Genetic and phenotypic analyses provide further evidence implicating both genes in the regulation of meiosis. Phenotypic analysis of sexual development showed that Znf3 is required for hyphal development during unisexual reproduction and also plays a central role during bisexual reproduction. Znf3 promotes cell fusion and pheromone production through a pathway parallel to and independent of the pheromone signaling cascade. Surprisingly, Znf3 participates in transposon silencing during unisexual reproduction and may serve as a link between RNAi silencing and sexual development. Our studies illustrate the power of unbiased genetic screens to reveal both novel and conserved circuits that operate sexual reproduction.

## Author Summary

Sexual reproduction drives genetic diversity throughout the eukaryotic kingdom and also purges deleterious mutations. Sexual development usually occurs between partners of opposite sex or mating type; however, in the absence of a compatible partner, unisexual reproduction can occur involving cells of only one mating type in some fungal species. The human fungal pathogen *Cryptococcus neoformans* undergoes a dimorphic switch during bisexual and unisexual reproduction. How genetic circuits control and distinguish these developmental cascades was unknown. We employed a genome-wide insertional mutagenesis approach combined with transcriptional profiling to identify three novel factors that affect different stages of hyphal development during bisexual and unisexual reproduction. Znf3 orchestrates hyphal development during sexual reproduction, and Spo11 and Ubc5 are required for the production of viable meiotic spore progeny. The findings presented here illustrate the complexity of genetic circuits that govern the two distinct modes of sexual reproduction and offer a foundation to further elucidate the communication and interaction between the molecular pathways.

## Introduction

Sexual reproduction in eukaryotes facilities genetic diversity and eliminates deleterious mutations leading to better fit progeny. In fungi, sex often involves two cells of opposite mating type (heterothallism) that secrete pheromones in order to induce cell fusion and subsequently nuclear fusion and meiosis generate recombinant progeny. However, in other fungi, solo incubation of an individual isolate can result in sexual reproduction, and this selfing process is referred to as homothallism. Homothallism can involve 1) mating type switching, 2) the presence of both mating type alleles (fused or unlinked), or 3) unisexual reproduction of just one mating type [1]. Paradigmatic examples of fungi with both modes of sexual reproduction are *Saccharomyces cerevisiae* and *Cryptococcus neoformans*.

*S. cerevisiae* has served as a model for the exploration and elucidation of molecular mechanisms of the dimorphic switch and therefore serves as a framework for morphogenesis in other dimorphic fungi [2]. Pheromones produced by the opposite-mating type partner activate the mitogen-activated protein kinase (MAPK) signaling cascade (which is also called the pheromone response pathway) to induce mating, pseudohyphae, and invasive growth [3], [4]. Additional environmental cues are known to regulate pseudohyphae formation. Nutrients activate cellular receptors and the cAMP-dependent pathway to govern the expression of genes evoking the dimorphic transition [5]. Core components of these pathways are highly conserved throughout the fungal kingdom; however, the downstream targets are often species-specific.

*Cryptococcus neoformans* is a human fungal pathogen that grows as a yeast in the environment and inside the host. It causes severe central nervous system infections in HIV-infected patients and less frequently in immunocompetent individuals. Cryptococcal meningitis is an AIDS-defining illness in 30% of HIV infections worldwide and is uniformly fatal if untreated [6].

*C. neoformans* yeast cells undergo a dimorphic switch into hyphae during sexual development. The organism has a defined life cycle with α (common) and **a** (rare) mating type cells. Under nutrient-limiting conditions or in response to inositol, cells of the opposite mating type secrete pheromones leading to cell-cell fusion [7], [8], [9], [10]. The two cells fuse to form a stable heterokaryon, which undergoes the dimorphic switch to filamentous growth. The two haploid nuclei remain separate in the growing hyphae and migrate from one hyphal cell to the adjacent one through clamp cells that fuse to connect the neighboring hyphal cells. At the apex of the aerial filaments, specialized, swollen structures known as basidia form where nuclear fusion and meiosis occur [8]. Multiple rounds of mitosis and budding produce four chains of spores, which are dispersed by air and germinate to re-enter the life cycle [11]. The dimorphic switch during bisexual reproduction occurs in both the A and D serotypes of *C. neoformans*, as well as in the sibling species *C. gattii* [8], [12], [13], [14]. Meiosis and sporulation are integral to the *C. neoformans* life cycle and play important roles in virulence. Cryptococcal infections are acquired by the inhalation of infectious particles, spores or desiccated yeast cells, from the environment. Spores are of an ideal size to penetrate and colonize the alveoli of the lung and are pathogenic [15], [16].

The predominance of the α mating type in all varieties of *Cryptococcus* and the presence of highly clonal populations led to the hypothesis that this pathogen might have a predominantly asexual life cycle. However, previous studies have shown that the dimorphic switch can occur during unisexual reproduction involving cells of only one mating type [17], [18]. Unisexual reproduction has been directly observed under laboratory conditions mainly in the serotype D lineage. In response to nutrient limitation, α cells of a single haploid isolate grown in solo culture can form a haploid or a diploid monokaryotic hyphae. Like bisexual reproduction (which can produce either a dikaryotic mycelium with paired haploid nuclei or a diploid monokaryotic mycelium [19]), during unisexual reproduction the haploid nuclei can either diploidize early or later in the basidium just prior to meiosis (Figure S1). Diploidization can result from either cell-cell and then nuclear fusion, or via endoreplication. The resulting hyphae grow to form basidia where meiosis and repeated rounds of mitosis and budding occur to produce haploid meiotic progeny as long chains of spores [18]. That the meiotic recombinase Dmc1 is required for spore production and germination shows definitively that unisexual reproduction is a meiotic process [18]. *Candida albicans* also has been found to undergo unisexual reproduction in the absence the of Bar1 protease, or in ménage à trois matings [20], similar to α-α cell fusion stimulated by **a** cells in *Cryptococcus* [18]. Unisex may serve to introduce genetic diversity and confer adaptive benefits.

The MAPK pheromone response signaling cascade governs dimorphism during bisexual and unisexual reproduction in *C. neoformans*. This signaling circuit is structurally and functionally conserved among both closely (i.e. *Ustilago maydis*) and distantly related fungi (i.e. *S. cerevisiae*). The pathway is activated by pheromones and orchestrates signal transduction through sequential phosphorylations evoked by the Ste20α/**a** (p21-activated kinase, PAK), Ste11α/**a** (MAPK kinase kinase), Ste7 (MAPK kinase), and Cpk1 (MAPK) kinases [21], [22]. Deletion of the corresponding genes severely impairs hyphal development during sexual development [21], [23]. In *S. cerevisiae*, the main transcriptional target of the pathway is Ste12, and a conserved *STE12*α/**a** homolog is present in the mating-type locus of *C. neoformans* [24], [25]. Recent studies have shown that the MAPK pathway target is Mat2, an HMG protein essential for bisexual and unisexual reproduction [26], that binds Pheromone Response Elements (PRE) in the promoters of the pheromone genes and 11 other pheromone induced genes [27]. A conserved cAMP pathway and a MAPK signaling cascade govern mating, morphogenesis, and pathogenicity in the closely related basidiomycete *Ustilago maydis*. However, the downstream transcription factor is a distinct HMG protein, Prf1, which regulates virulence and is pheromone-induced [28]. Thus the molecular pathway governing sexual development has been rewired since the divergence of both *U. maydis* and *C. neoformans* from their last common ancestor shared with *S. cerevisiae*.

While **a**-α bisexual reproduction and α-α unisexual reproduction are related sexual cycles, there are distinguishing characteristics. Notably, the cell identity homeodomain Sxi1α/Sxi2**a** protein complex is specifically required for bisexual mating but dispensable for unisexual reproduction [18]. Other distinct elements that may specifically regulate the two sexual cycles, and which remained to be identified involve hyphal elongation, basidia formation, meiosis, and sporulation.

The aim of this study was to identify and characterize unique regulators of unisexual reproduction. We employed a high-throughput genetic screen using an *Agrobacterium*-transkingdom DNA delivery approach to isolate random insertional mutants [26], [29], [30], [31] in a genome sequenced self-fertile haploid strain and isolates with filamentation defects were identified by microscopy. We disrupted genes encoding three regulators of unisexual development: Spo11, Ubc5, and Znf3. Spo11 is the homolog of *S. cerevisiae* Spo11, which induces DNA double-strand breaks (DSBs) that promote meiotic recombination [32]. *UBC5* encodes a ubiquitin-conjugating enzyme that degrades short-lived or abnormal proteins in *S. cerevisiae* and also has a role in sporulation [33]. Deletion of either the *SPO11* or the *UBC5* gene significantly reduced spore number and germination providing additional definitive evidence, beyond the established role of Dmc1, that *Cryptococcus* unisexual reproduction is a meiotic process. Znf3 is a novel zinc finger protein. Phenotypic analysis of Znf3 insertion and deletion mutants showed that this component regulates hyphal development during both bisexual and unisexual reproduction. *znf3* mutations impaired cell-cell fusion and blocked hyphal development, indicating a role in the early steps of sexual reproduction. Based on epistasis analysis, Znf3 is not a transcriptional target of the pheromone signaling cascade, but rather contributes to modulate Mat2 expression and is required for pheromone expression. Surprisingly, deletion of *ZNF3* increased expression of transposons and transposon-related genes during unisexual reproduction. These results support the conclusion that Znf3 regulates sexual development through a different circuit parallel to the pheromone response pathway and governs pheromone gene expression and silences transposons, either directly or indirectly. Taken together these results proffer new insights into the control of hyphal development during bisexual and unisexual reproduction.

## Results

### Mutants altered for self-filamentous unisexual reproduction

To identify genes encoding novel components governing unisexual reproduction we utilized the ability of *A. tumefaciens* to transfer and mutagenize its target by randomly introducing a known DNA sequence into the genome. *A. tumefaciens*-mediated transkingdom DNA delivery has been successfully applied in *C. neoformans* to identify and characterize novel virulence factors and components of other sensing pathways [26], [30], [31], [34]. Similar *Agrobacterium*-mutagenesis studies by Lin *et al.* investigated the transition from yeast to hyphae with a limited number of mutants and identified Mat2, the major transcriptional regulator of the pheromone response signaling cascade [26] whereas Fu *et al.* identified the scaffold protein Ste50 [35].

Here, we sought to dissect each of the sequential steps of unisexual reproduction. A large library of insertion mutants was screened to identify and characterize genes required for hyphal initiation, hyphal elongation, basidia formation, and spore production. We utilized the haploid serotype D strain XL280α that, when solo cultured on appropriate media (V8, MS, FA, SLAD), undergoes robust hyphal development via unisexual reproduction [36]. The nuclear content of the vegetative yeast cells budded from the hyphae (blastospores) reflect the ploidy of the hyphae. XL280 generates a mixture of diploid and haploid hyphae during unisexual reproduction based on FACS (Fluorescent-Activated Cell Sorting) analysis in two independent experiments (Figure S1). Diploid blastospores [5/19 (26%) and 9/24 (37.5%) hyphae examined] are produced by budding from a diploid monokaryotic hyphae that is generated by early nuclear diploidization. In the haploid monokaryotic hyphae [14/19 (74%) and 15/24 (62.5%) hyphae examined] late diploidization occurs in the basidium to generate a diploid nucleus ([37]. In both cases the homozygous diploid nucleus undergoes meiosis and sporulation to produce the basidiospores that form four chains of spores on the surface of the basidium. Ploidy was congruent in all cases with hyphal behavior: diploids were hyperfilamentous compared to haploids.

XL280α is a laboratory strain derived from a cross of two sibling strains, JEC20**a** (congenic with JEC21α) and B3501α, which share ∼50% genome identity. Both of the parental strains have been sequenced [38]. Whole genome sequence analysis of XL280α reveals that it shares ∼81% genome identity with the congenic strains JEC20**a** and JEC21α (Ni, Feretzaki, Li, Floyd-Averette, Mieczkowski, Dietrich, and Heitman, submitted to *PLOS Biology*).

*A. tumefaciens* mediated insertional mutagenesis generated approximately 6,100 mutants yielding ∼1× genomic coverage. The mutants were transferred onto unisex-inducing V8 medium and incubated at room temperature in the dark for 14 days. The mutant library was then screened microscopically for enhanced or diminished hyphal development during unisexual reproduction. The library was also incubated on V8 medium for 14 days under constant light conditions. Light inhibits sexual development in *Cryptococcus* and this process is genetically regulated by the two light sensing genes *BWC1* and *BWC2* [30], [39]. To identify candidate genes for light sensing, we also screened the library for mutants that filamented equally well in the dark and the light.

**Figure 1:**
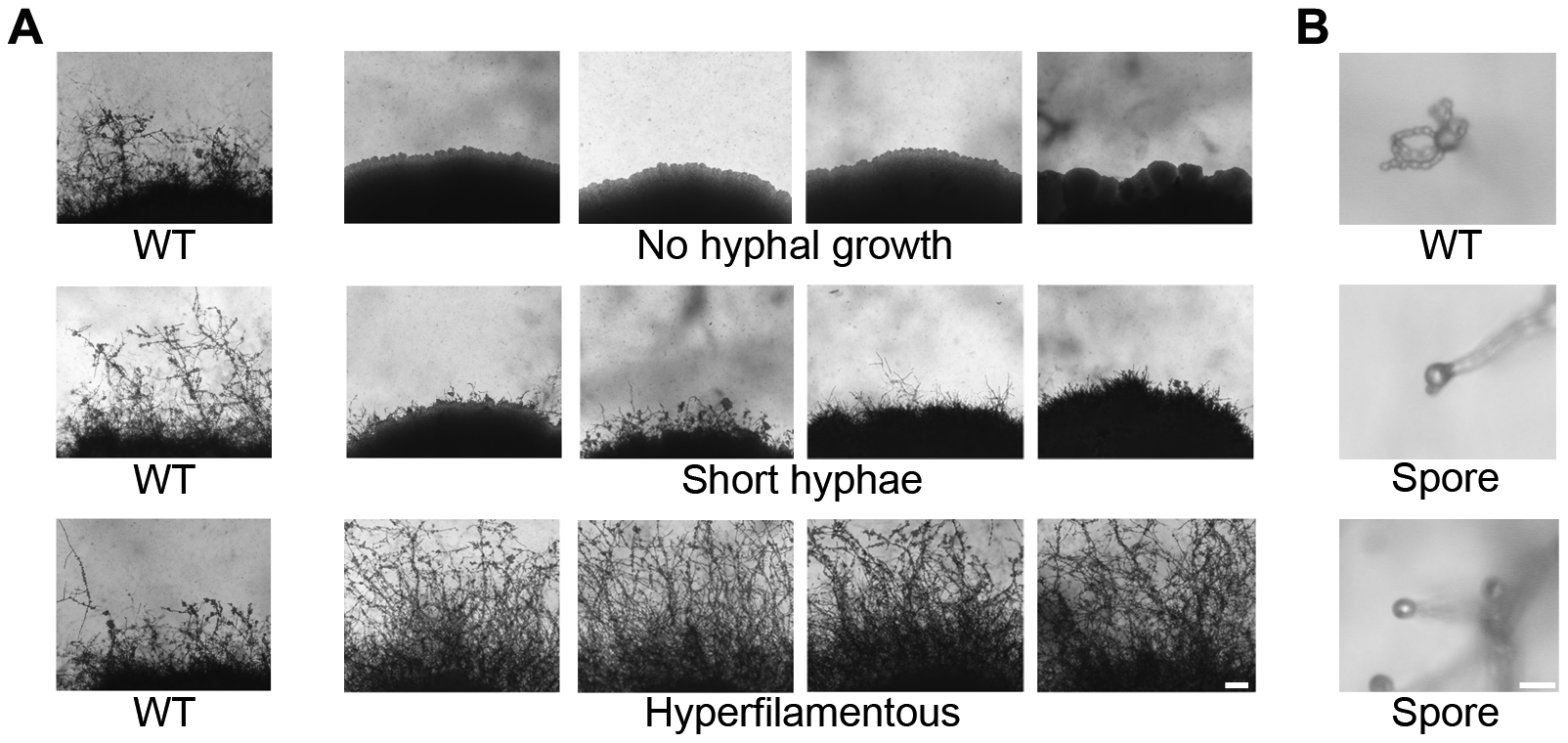
Phenotypic analysis of insertion mutants altered in unisexual reproduction. Nourseothricin (NAT)-resistant *Agrobacterium* transconjugant mutants of XL280α were grown on V8 solid agar for 14 days in the dark at room temperature to examine hyphal development during sexual reproduction by light microscopy. (**A**) The wild type strain XL280α undergoes robust hyphal growth during unisexual reproduction. The most extreme mutant phenotypes include no hyphal development, short hyphae, or hyperfilamentous strains. The scale bar represents 100 µm. (**B**) The progeny of wild type strain XL280α are organized in four long chains of spores that emerge from the surface of the basidium. Sporulation was severely impaired in several insertion mutants. The scale bar represents 10 µm.

We identified 225 mutants that exhibited a stable filamentation difference resulting in no hyphal growth (16), short hyphae (131), abnormal hyphae (21), more abundant hyphae (46), sporulation defects (2), increased chlamydospore production (6), or altered light response (3) (Figure 1). Mutants with sporulation defects and no hyphal growth or a hyperfilamentous phenotype were subjected to PCR to identify insertions in known candidate genes. Our screen was successful in isolating mutations in the previously identified genes *CRG1*, *STE7*, *MAT2* (two independent mutants), and *ZNF2*, whose products are key components of the pheromone signaling cascade and in *BWC2*, which encodes a major regulator of light sensing [13], [21], [26], [30]. Isolates with increased ploidy (2N diploid or 3N triploid) are known to exhibit more robust filamentation during unisexual reproduction compared to congenic haploid strains [18]. All of the hyperfilamentous mutants were subjected to FACS analysis to determine their ploidy state, and four hyperfilamentous mutants were found to be diploid (2N). Using a microdissection microscope, 20 basidiospores were isolated from each diploid isolate; hyphal development of all of these progeny was similar to the wild type XL280α parental strain. FACS analysis of 15 progeny from each diploid showed that they were all haploid, indicating that their hyperfilamentous phenotype was due to an increase in ploidy (2N) (and not to a mutation enhancing diploidization). These findings further confirm that 1) α/α diploid isolates undergo unisexual reproduction to produce haploid progeny and 2) diploid spore isolates can be recovered from the sexual cycle (see also [19]).

To identify insertions in unknown genes, the mutants were subjected to linkage analysis, inverse PCR, and sequence analysis. Linkage analysis of the mutants identified 12 in which the insertion was not linked with the mutant phenotype and these were excluded from further characterization. Of the remaining mutants 31 yielded an inverse PCR product. The sequences obtained were used in BLAST searches against the *C. neoformans* JEC21 genome database to identify the disrupted genes. Table 1 summarizes the insertion sites for 31 mutants. One of the newly identified genes from mutants that were afilamentous, CNK01880, encodes a protein with three zinc finger domains (Text S1, Figure S2). Zinc finger proteins are often involved in binding to nucleic acids (DNA and RNA), and the C2H2 motif is often a feature of transcription factors. The presence of three C2H2 zinc finger domains prompted us to name this protein Znf3 to distinguish it from two other genes encoding zinc finger transcription factors (Znf1 and Znf2) [26], [40]. Two sporulation defective mutants had insertions in two highly conserved genes. The first gene, CNH01330, encodes a topoisomerase homolog and the second, CND02770, encodes a ubiquitin-conjugating enzyme (see Text S1, Figure S2). Because these genes are the orthologs of the *S. cerevisiae* meiosis-specific endonuclease Spo11 and the ubiquitin-conjugating enzyme Ubc5, we named the *Cryptococcus* genes *SPO11* and *UBC5*, respectively.

**Table 1:** Genes governing unisexual reproduction.

Genbank Accesion	Protein class/Identifiable motifs^c^	Gene disrupted	Close homolog/Known role
**No hyphal growth**			
CNM02020	HMG domain at N-terminus	*MAT2* a ^,^ b	Sexual development transcription factor
CNE04290	Rab-GTPase activation domain at N-terminus		**Gyp5**, polarized growth in *S. cerevisiae*
CNF02400	Cell wall and biogenesis related protein	*SLA2* b	**Sla2**, cytoskeleton factor in *S. cerevisiae*
CNK01880	Two C2H2-type zinc finger domains	*ZNF3* a ^.^ b	
CNC02350	Mitogen activating protein kinase kinase	*STE7* a	MAP kinase kinase mating factor
CNF04390	Velvet-like factor	*VEA2* a	Velvet factor **VeA** in *Aspergillus*
CNG02160	Two C2H2-type zinc finger domains	*ZNF2* a	Mammalian **Znf2.2** factor
**Hyperfilamentous growth**			
CNC02080	Rta1-like protein		Lipid-translocating exporter in *S. cerevisiae*
CNG01130	NAD epimerase		Highly conserved eukaryotic protein
CNB02170	Transmembrane efflux protein		**EncT** ergosterol transport in *C. neoformans*
CNI00820	Histidine phosphatase domain of mutases		Highly conserved eukaryotic protein
CNJ01220	Hypothetical protein		Conserved in Tremellaceae protein
CNA03100	single-stranded DNA endodeoxyribonuclease		Highly conserved eukaryotic protein **Rad2**
CNC01470	GTP-binding protein		Highly conserved GTPase protein
CNC03490	Leucine rich repeats		Septation initiation scaffold **Cdc11** *S. pombe*
CNA02470	Fungal gamma tubulin protein		**Spc97** microtubule complex in *S. cerevisiae*
CNC02700	Mitochondrion organization-related protein		Homolog of human **Letm1**
CNG04300	3-hydroxyacyl-CoA dehydrogenase		Highly conserved eukaryotic protein
CNF04140	Rho small monomeric GTPase		**Rho2**, involved in cell polarity in *S. cerevisiae*
CNG03390	Yippee-like putative protein		Highly conserved eukaryotic protein
CNH01390	Fungal specific transcription factor		Highly conserved fungal protein
CNH02070	Serine/threonine protein kinase		**Kin4**, inhibits mitotic exit network in yeast
CNC02950	Hypothetical protein		Conserved in Tremellales
CNA01150	Regulator of G protein signaling domain	*CRG1* a	Crg1 negative regulator of mating in *C. neoformans*
CNK02180	GMP synthase		**Gua1**, negatively regulated by nutrient starvation in *S. cerevisiae*
CNG04410	Hypothetical protein		Basidiomycota conserved protein
CNL03660	Hypothetical protein		Sga1, glycogen starvation in *S. cerevisiae*
**Sporulation defect**			
CND02770	Ubiquitin-conjugating enzyme E2-domain	*UBC5* a	**Ubc5**, Ubiquitin-conjugating enzyme in *S. cerevisiae*
CNH01330	Type IIB DNA topoisomerase VI	*SPO11* a	**Spo11**, catalyzes DSB in *S. cerevisiae*
**Light sensing defect**			
CNE01220	ZnF-GATA and PAS domains	*BWC2* a	**Wc1**, role in light sensing and circadian rhythm in *N. crassa*
CNJ01160	Arylsulfotransferase	*BWC3* a ^,^ b	Highly conserved eukaryotic protein

Linkage analysis showed that the insertions in these mutants are linked to the phenotype.

a *De novo* gene disruption confirmed the mutant phenotype.

b Multiple independent insertion mutants were isolated.

c Conserved domains were identified using the InterProScan Sequence Search tool of EMBL-EBI (http://www.ebi.ac.uk/Tools/pfa/iprscan/).

### Znf3 is essential for unisexual reproduction

**Figure 2:**
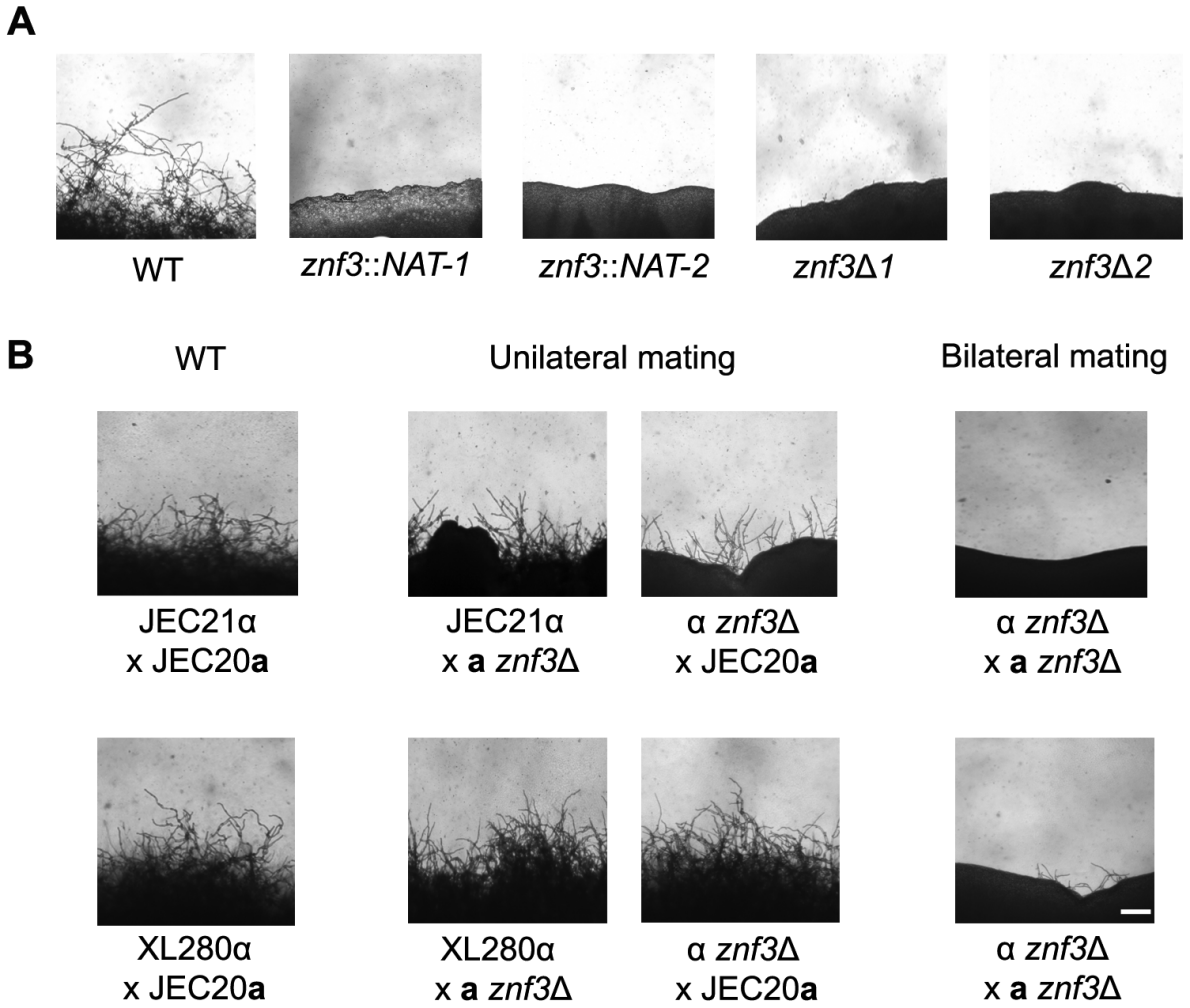
Deletion of *ZNF3* impairs hyphal development during bisexual and unisexual reproduction. (**A**) XL280α, two mutants with independent insertions in *ZNF3* (II-12, II-147), and two independent *znf3*Δ deletion mutants (MF01, MF38) were incubated on V8 medium in the dark at room temperature for 10 days and hyphal formation during unisexual reproduction was assessed by microscopy. (**B**) Bisexual wild type crosses (JEC21α×JEC20**a** and XL280α×JEC20**a**), unilateral mutant crosses (α *znf3*Δ×WT**a** and WTα×**a** *znf3*Δ), and bilateral mutant crosses (α *znf3*Δ×**a** *znf3*Δ) were conducted on V8 medium in the dark at room temperature for 7 days and photographed. The scale bar represents 100 µm.

The genetic screen revealed five different mutants with a similar filamentation defect, each with an insertion into a different site in the *ZNF3* gene. One insertion was in the coding region of the gene, and four other insertions were in different positions of the promoter region. Insertion in promoters or terminators of a gene is a common feature of T-DNA insertions, which exhibit a bias towards promoters and 5′ and 3′ UTRs [41]. To elucidate the role of Znf3 in unisexual reproduction, we deleted the complete gene, from start to stop codon, in XL280α via biolistic transformation and homologous recombination. Two independent *znf3*Δ deletion mutants were generated, and the deletions were verified by Southern blot and PCR analyses. We assessed the ability of the insertion and deletion mutants to undergo unisexual reproduction by incubating the strains in the dark for two weeks on different filamentation-inducing media and examining them by light microscopy. The wild type XL280α strain formed hyphae along the entire periphery of the colony, while two *znf3* insertion mutants were defective in hyphal production (Figure 2A). The *znf3*Δ mutation blocked hyphal development in both independent mutants (Figure 2A). Prolonged incubation on filamentation inducing media (≥3 weeks) did not result in the production of any hyphae, indicating that Znf3 is critical for the dimorphic transition during unisexual reproduction.

### Znf3 plays a role in hyphal development during bisexual reproduction

Because the pheromone signaling cascade components are known to regulate both bisexual and unisexual reproduction [21], we hypothesized that Znf3 might also play a role in the dimorphic transition during bisexual reproduction. As mentioned earlier, the **a**-α sexual cycle is induced by the fusion of opposite-mating type cells to form a dikaryon, which initiates the development of dikaryotic hyphae and leads to the formation of basidia, where nuclear fusion and meiosis occur to produce chains of spores. To determine the role of Znf3 in bisexual reproduction, we deleted the gene in both strains of the serotype D congenic mating pair JEC21α and JEC20**a** via biolistic transformation. Because two different mating type partners are involved we used unilateral (one mating partner is mutant) and bilateral (both mating partners are mutant) mating assays to examine the role of Znf3. In the wild type JEC21α×JEC20**a** cross, filamentation was robust, with basidia and spores forming after 7 days of incubation on V8 medium. However, unilateral crosses of *znf3*Δ (α *znf3*Δ×WT or WT×**a** *znf3*Δ) produced significantly fewer hyphae, while in bilateral crosses (α *znf3*Δ×**a** *znf3*Δ), hyphal development was severely impaired (Figure 2B). Unilateral mutant crosses were able to complete the sexual cycle and form spores following prolonged incubation on V8 (∼2 weeks), while bilateral mutant crosses produced only sporadic hyphae. In contrast, the *znf3*Δ mutant phenotype was less severe in the hyperfilamentous XL280α background. Unilateral matings exhibited hyphae with basidia and spores at approximately wild type levels (Figure 2B). However, hyphal development was impaired in bilateral matings with sporadic short hyphae that eventually grew long enough to produce basidia and spores (∼3 weeks). Based on these findings, we conclude that Znf3 plays an important role during **a**-α mating.

Defects in hyphal development could be due to defects in fusion of α and **a** mating partners. Because the *znf3*Δ mutation blocked the dimorphic transition during bisexual reproduction, Znf3 could be involved in the initial step of mating. Cell-cell fusion assays were employed to determine the role of *ZNF3* in cell-cell fusion. Wild type α and **a** strains and α *znf3Δ* and **a** *znf3Δ* mutant strains genetically marked with different drug resistance markers were mated on V8 medium for 15 or 24 hours in the dark. The cells were collected and plated in serial dilutions on media that select for doubly drug-resistant fusion products (to measure cell-cell fusion) or allow all cells to grow (to determine total CFU). In *znf3*Δ×WT unilateral matings, the relative fusion efficiency was ∼50% at 15 hours of co-incubation and ∼70% at 24 hours compared to wild type. Interestingly, in *znf3*Δ×*znf3*Δ bilateral matings, the fusion efficiency was reduced to ∼15% after 15 hours of co-incubation and ∼50% after 24 hours of co-incubation. In contrast, disruption of the *CPK1* MAPK and *MAT2* transcription factor genes essentially abolished cell-cell fusion in both unilateral and bilateral mating assays, indicating an essential role in the first steps of sexual reproduction [21], [26]. These observations support the hypothesis that Znf3 plays a modest role in cell-cell fusion events early in mating. However, with prolonged incubation times, *znf3Δ* mutant cells do undergo efficient cell-cell fusion. A possible explanation could be that Znf3 acts in parallel with the pheromone signaling cascade and that it is required to enhance the intracellular signal for the dimorphic transition during bisexual reproduction.

### Znf3 regulates pheromone production

The dimorphic transition during bisexual and unisexual reproduction is in part induced by the production of peptide pheromones. The Cpk1 MAPK signaling cascade mediates sensing and pheromone production during sexual development [21], [26]. To determine whether Znf3 plays a role in the pheromone-sensing pathway, we generated expression profiles for the *ZNF3*, *MAT2*, *SXI1*α, and *MF*α*1* genes in wild type, *ste7*Δ, *mat2*Δ, *znf2*Δ, *sxi1*αΔ, and *znf3*Δ mutant backgrounds. Gene expression was monitored during bisexual and unisexual reproduction by RT-PCR. We determined that expression of these genes in wild type bisexual reproduction reached the highest levels after 24 hours of incubation on filamentation-inducing V8 medium (Figure S3). *MF*α*1* expression, which increased ∼2,500-fold, served as a positive control. During unisexual reproduction, expression of the genes in XL280α reached the highest level after 48 hours of incubation on V8. These time points were chosen to determine the expression of the genes in the different mutant backgrounds.

**Figure 3:**
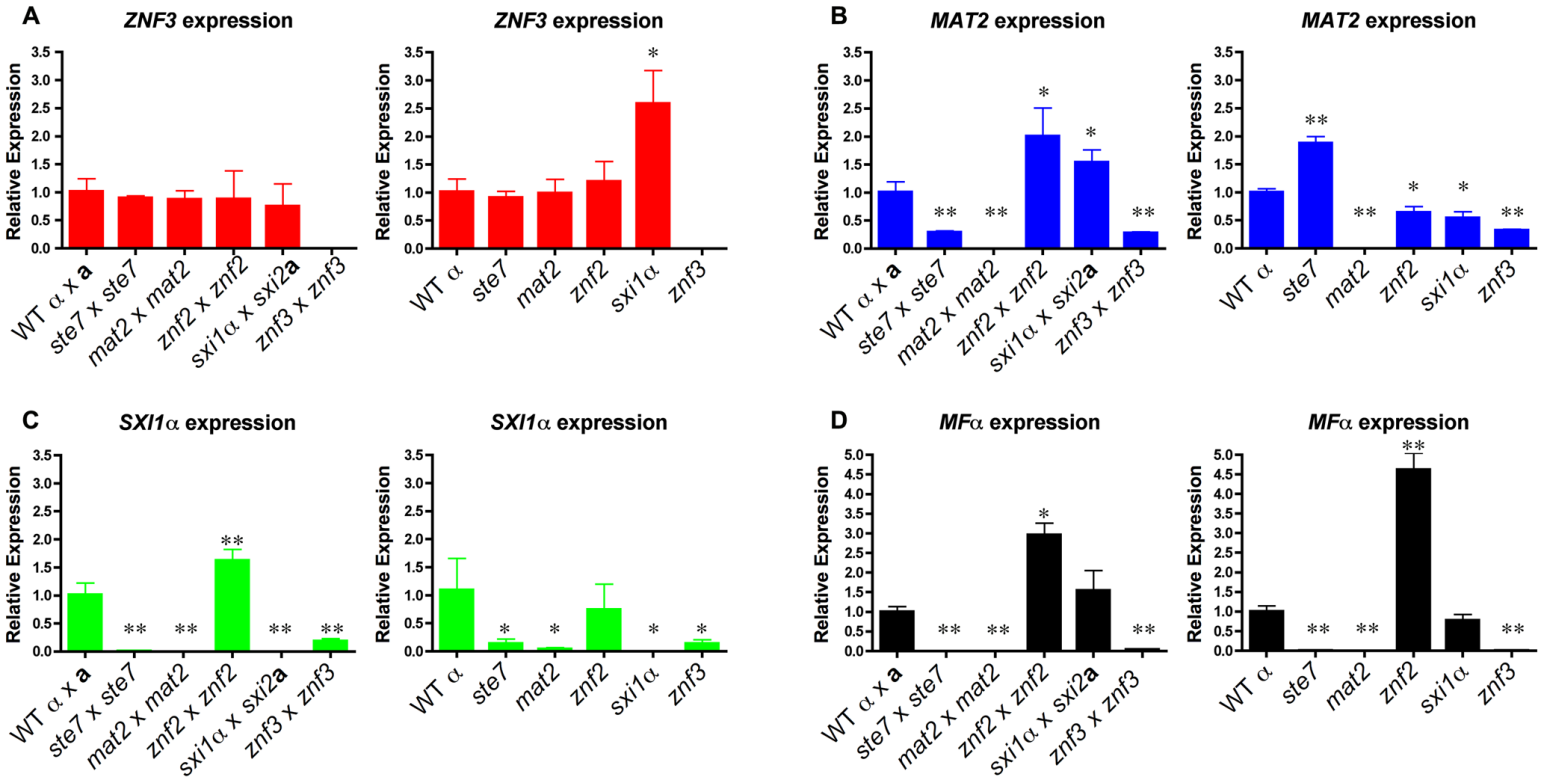
Znf3 regulates the expression of Mat2 and promotes pheromone production. Cells were incubated for 24 hours on V8 medium for bisexual and 48 hours for unisexual reproduction. Yeast and hyphal cells were harvested and RNA was isolated. The expression of the (**A**) *ZNF3*, (**B**) *MAT2*, (**C**) *SXI1*α, and (**D**) *MF*α*1* genes was measured by RT-PCR in wild type, *ste7*Δ, *mat2*Δ, *znf2*Δ, *sxi1*αΔ, and *znf3*Δ mutants (* indicates P<0.05 and ** indicates P<0.005 compared to the WT). The error bars represent the standard deviations from the mean for the three biological replicates.

Both bilateral bisexual and unisexual reproduction were performed on V8 media and the cells were harvested at the designated times. RNA was isolated, and RT-PCR was employed to determine the transcript levels in each mutant. *ZNF3* expression remained the same and similar to wild type, even when components of the MAPK signaling cascade were absent during crosses (Figure 3A). This indicates that Znf3 is not a transcriptional target of the pheromone-signaling cascade and that expression does not depend on the MAPK pathway. However, during bisexual reproduction, *MAT2* expression was decreased in the *znf3*Δ mutant in an expression pattern similar to that of *ste7*Δ mutants previously observed (Figure 3B) [26]. The *ste7*Δ phenotype is in accord with the hypothesis that Mat2 is the direct target of the MAPK signaling cascade; however, Znf3 may moderately co-regulate the expression levels of Mat2 during hyphal development, and in fact *MAT2* expression was also decreased in the *znf3*Δ mutant during unisexual reproduction. A similar phenotype was observed for Sxi1α. Previous studies have shown that Mat2 governs *SXI1*α through the Cpk1 MAPK cascade in bisexual reproduction [26]. *SXI1*α expression was abolished in *ste7*Δ and *mat2*Δ mutant backgrounds. Deletion of *ZNF3* significantly decreased the transcript levels of *SXI1*α compared to wild type; however, the level of inhibition was less severe than in the *ste7*Δ and *mat2*Δ mutant backgrounds (Figure 3C). In unisexual reproduction, the *znf3*Δ mutant decreased *SXI1*α expression similar to *ste7*Δ and *mat2*Δ mutants (Figure 3C). Interestingly, the inhibition profile of *SXI1*α during bisexual reproduction was similar to unisexual reproduction, even though *SXI1*α does not participate in hyphal development during unisex.

In further experiments, we determined the expression levels of other components of the MAPK signaling cascade. During bisexual reproduction the transcriptional profile of *CPK1* was similar to *MAT2* with modestly reduced levels in *znf3*Δ bilateral crossing cultures (Figure S4A). We also assessed the expression levels of two negative regulators of the pheromone pathway, *CRG1* and *CRG2*, in the absence of components of the MAPK signaling cascade during unisexual reproduction (Figure S4B). *CRG2* expression levels were slightly reduced in the deletion mutants. *CRG1* transcription significantly increased in the *znf2*Δ mutant, which may contribute to the inhibition of hyphal development. However, expression of these negative regulators was not significantly altered in the *znf3*Δ mutant.

Pheromones are key activators of sexual development, as well as important targets. During vegetative growth the pheromones are expressed at basal levels and in a nutrient-limited environment the pathway is activated by a massive increase in pheromone production enforced by a positive feedback loop. Deletion of components of the Cpk1 MAPK cascade blocks expression of the pheromones and inhibits responses [21], [26]. By measuring the transcript levels of *MF*α*1* in bilateral bisexual and unisexual reproduction in *ste7*Δ and *mat2*Δ mutant backgrounds, we found that the inducible expression level was completely blocked compared to the wild type (Figure 3D). In contrast, Znf2 functions further downstream and regulates hyphal formation without participating in the initiation of the pheromone signal [26]. *MF*α*1* expression in the *znf2*Δ mutants was significantly increased in both sexual cycles, as previously observed (Figure 3D) [26]. On the other hand, the *sxi1*αΔ mutation modestly increased pheromone expression during bisexual reproduction as previously observed [42]. However, *MF*α*1* production was significantly reduced in the *znf3*Δ mutant. During both bisexual and unisexual reproduction of *znf3*Δ mutants the expression of the pheromone gene was severely impaired (Figure 3D). This indicates that Znf3 is important for pheromone production, and the hyphal defect of *znf3*Δ mutants may result in part from impaired *MF*α*1* expression. Nevertheless, the expression of the pheromone was not completely blocked and the expression profile pattern was similar to *MAT2* and *SXI1*α expression in the *znf3*Δ mutant. Taking these results together, we can conclude that Znf3 is not regulated by the pheromone-signaling cascade; however, it may act in a parallel pathway that regulates the expression of the pheromone or Mat2 and consequently its targets, including Sxi1α.

### Znf3 governs sexual reproduction through an independent pathway

**Figure 4:**
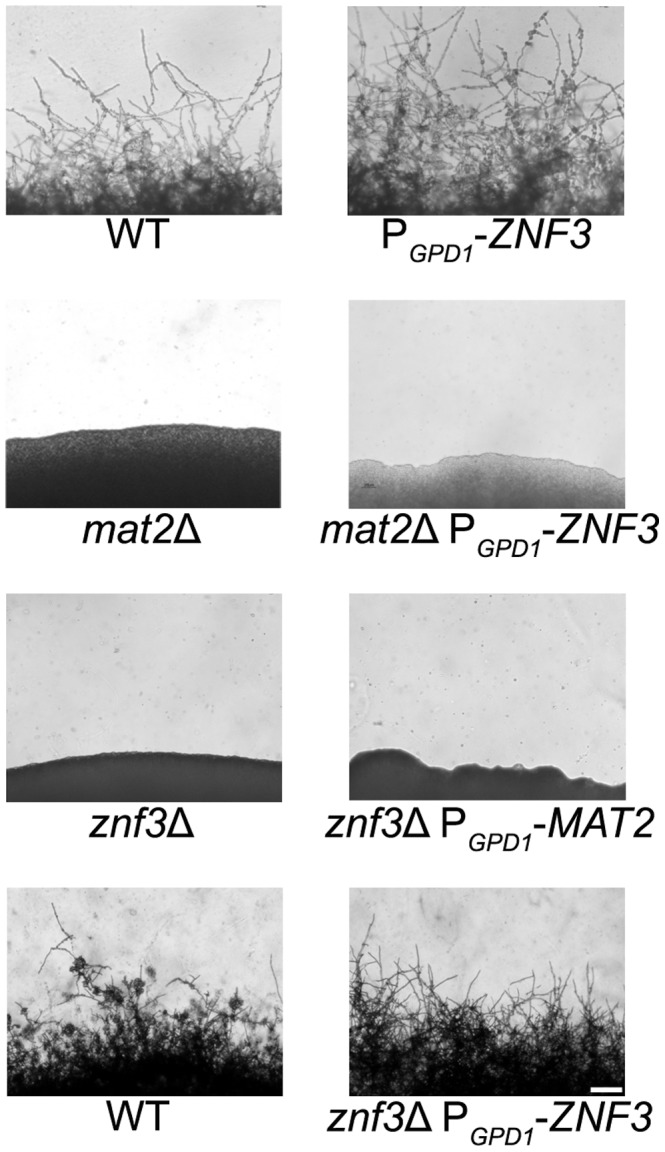
Znf3 is not a transcriptional target of the MAPK signaling cascade or Mat2. Wild type XL280α, *mat2*Δ, and *znf3*Δ mutants were transformed with the P*_GPD1_*-*ZNF3* transgene, and a *znf3*Δ mutant was transformed with the P*_GPD1_*-*MAT2* transgene. The cells were incubated on V8 medium for 14 days in the dark. The *ZNF3* overexpression allele moderately increased hyphal development in the wild type and restored unisexual reproduction in the *znf3*Δ mutant; however, it failed to suppress the sterile phenotype of *mat2*Δ mutants. Similarly, the *MAT2* overexpression allele failed to restore hyphal development of the *znf3*Δ mutant. The scale bar represents 100 µm.

Znf3 is required for hyphal growth during bisexual and unisexual reproduction, and we have shown that it plays an important role in pheromone expression. Although it is not a transcriptional target of the MAPK pathway, it may act downstream and contribute to or enhance the pheromone-induced signal. To investigate the possible role of Znf3 in the pheromone signaling cascade, we engineered an overexpression allele with *ZNF3* under the control of the constitutively active *GPD1* gene promoter. Wild type, a *mat2*Δ mutant, and a *znf3*Δ mutant were transformed with this overexpression allele using biolistic transformation. Based on RT-PCR results, expression of *ZNF3* was increased ∼5-fold in the otherwise wild type P*_GPD1_*-*ZNF3* background. Hyphal development was assessed on V8 medium by light microscopy. Hyphal growth in XL280α P*_GPD1_*-*ZNF3* was similar but slightly more robust compared to wild type (Figure 4). Because a wild type *ZNF3* complementation construct was not available, we transformed the *znf3*Δ mutant with the P*_GPD1_*-*ZNF3* allele and found that this overexpression allele restored unisexual reproduction (Figure 4). Hyphal growth was slightly more condensed in the complementation strain due to the ∼5 fold increased *ZNF3* expression, and it was moderately enhanced compared to the WT, possibly due to the expression from an exogenous locus.

Mat2 is a major component of the MAPK pathway, and overexpression of a downstream target should bypass its requirement for mating and stimulate hyphal production. However, overexpression of *ZNF3* failed to restore hyphal development in the afilamentous *mat2*Δ mutant, which indicates an independent function for Znf3 in unisexual reproduction (Figure 4). These observations provide further evidence that Znf3 does not act downstream of the pheromone signaling cascade.

Expression profiling showed that Znf3 moderately regulates the expression of *MAT2* in *C. neoformans*, indicating a mode of control on the major regulator of sexual reproduction. Transcriptional control of mating and morphogenesis in the closely related species *U. maydis* involves additional transcription factors upstream of Prf1, the HMG target of the conserved MAPK pathway [28], [43]. Constitutive expression of *PRF1* complements the severe defect caused by deletion of these factors. To determine if Znf3 functions upstream of Mat2 we used a *MAT2* allele under the control of the constitutively active *GPD1* promoter [26]. The overexpression *MAT2* allele was introduced into *znf3*Δ mutants via biolistic transformation and hyphal growth was assessed by light microscopy. Overexpression of *MAT2* in the *znf3*Δ background failed to induce the dimorphic switch from yeast to hyphae development (Figure 4). These results provide evidence that Znf3 and Mat2 may act in independent, parallel pathways. Transcription of *MAT2* is tightly regulated by the presence of pheromone, and during mating a 12-fold increase in expression was observed (Figure S3). Decreased expression of *MAT2* is possibly an indirect effect of the low pheromone levels observed in the *znf3*Δ mutant. The pathway that governs Znf3 and stimulates its control over pheromone expression warrants additional analysis.

### Znf3 regulates the expression of retrotransposons during sexual development

A lack of any obvious link between Znf3 and the pheromone-signaling cascade prompted us to analyze the genome-wide transcriptional profile of the *znf3*Δ mutant. We performed a comparative transcriptome analysis between XL280α and the *znf3*Δ and *mat2*Δ mutants during bisexual and unisexual reproduction. In the first level of analysis, we considered genes that were regulated similarly in the *znf3*Δ and *mat2*Δ mutants. As expected, the majority of the genes that were downregulated in the mutants were involved in the pheromone signaling cascade during unisexual reproduction. The expression of the pheromone and pheromone receptor genes was significantly decreased in both *znf3*Δ and *mat2*Δ mutants compared to the WT (Table S1). However, the whole genome transcriptional profile of the *znf3*Δ mutant was different (Figure S5). A similar phenotype was observed during bisexual reproduction (Table S1). This result validates our previous findings and supports the hypothesis that Znf3 acts in an independent, parallel pathway.

To determine the role of Znf3 during unisexual reproduction and obtain further mechanistic insights, we extended our genome-wide expression analysis to genes that are upregulated specifically in the *znf3*Δ mutant. More than 500 independent tags yielded more than a two-fold increase in the *znf3*Δ mutant during unisexual reproduction (Table S2). We aligned the highly enriched tags to the *C. neoformans* JEC21α genome database and found that the majority encoded putative transposases, hypothetical endonucleases, and RNA-dependent DNA polymerases (Table 2). Surprisingly, we also found a significant increase in the expression of the T1 and T3 DNA transposons (Table 2). Previous studies have shown that transposable elements are highly expressed during bisexual and unisexual reproduction; however, the RNAi pathway inhibits transposon activity via Sex-Induced Silencing (SIS) and protects the genome against transposition and mutations [44], [45], [46]. Deletion of *RDP1*, a component of the RNAi pathway (the RNA-dependent RNA polymerase), induces the expression of retrotransposons during sexual development [44], [46]. The previously identified expression profile of the *rdp1*Δ mutant exhibits a similar increase in transposable elements and RNA-dependent DNA polymerases as the *znf3*Δ mutant (Table 2). This type of regulation was not observed in the *mat2*Δ mutant, indicating a unique role for Znf3 in transposon silencing. Taking these results together, we conclude that Znf3 is required for transposon silencing and pheromone expression during sexual development, and these interesting findings will provide the foundation for further analysis in future investigations.

**Table 2:** Transposon-related genes are upregulated during *znf3*Δ mutant unisexual reproduction.

Gene Locus	*znf3*Δ/WT	Annotation
1712.seq.017	69.72	Transposable element T1
1671.seq.034	8.963	Hypothetical protein. Previously found to be upregulated in RNAi-mutant strains
1704.seq.045	8.76	Hypothetical protein. Previously found to be upregulated in RNAi- mutant strains
184.m05079	7.794	DDE Endonuclease, transposase domain
1661.seq.033	7.293	Hypothetical protein. Previously found to be upregulated in RNAi- mutant strains
1744.seq.140	7.231	Integrase, DDE superfamily endonuclease
177.m03075	7.188	DDE Endonuclease, transposase domain
183.m01854	6.929	Hypothetical protein. Previously found to be upregulated in RNAi- mutant strains
181.m08309	6.089	Transposable element T3
179.m00071	5.57	Transposable element T3
180.m00376	5.089	Transposable element T3
184.m04347	4.86	Transposable element T3
164.m02163	4.322	Transposable element T3
184.m05148	3.646	Transposable element T3
1642.seq.046	3.496	Transposable element T3
163.m06563	3.284	Transposable element T3
1631.seq.008	3.044	Transposable element T3
1663.seq.046	2.57	Transposable element T1
1681.seq.010	2.457	Transposable element T1
1682.seq.124	2.19	Transposable element T3
1742.seq.060	2.162	RNA-dependent DNA polymerase, present in transposable elements
1621.seq.158	2.102	Transposable element T3
163.m06565	2.096	Hypothetical transposase (Transposase family tnp2)
1751.seq.002	2.078	RNA-dependent DNA polymerase, present in transposable elements
1701.seq.190	2.018	Hypothetical protein, ATP-dependent DNA helicase domain

### Spo11 and Ubc5 are required for sporulation

**Figure 5:**
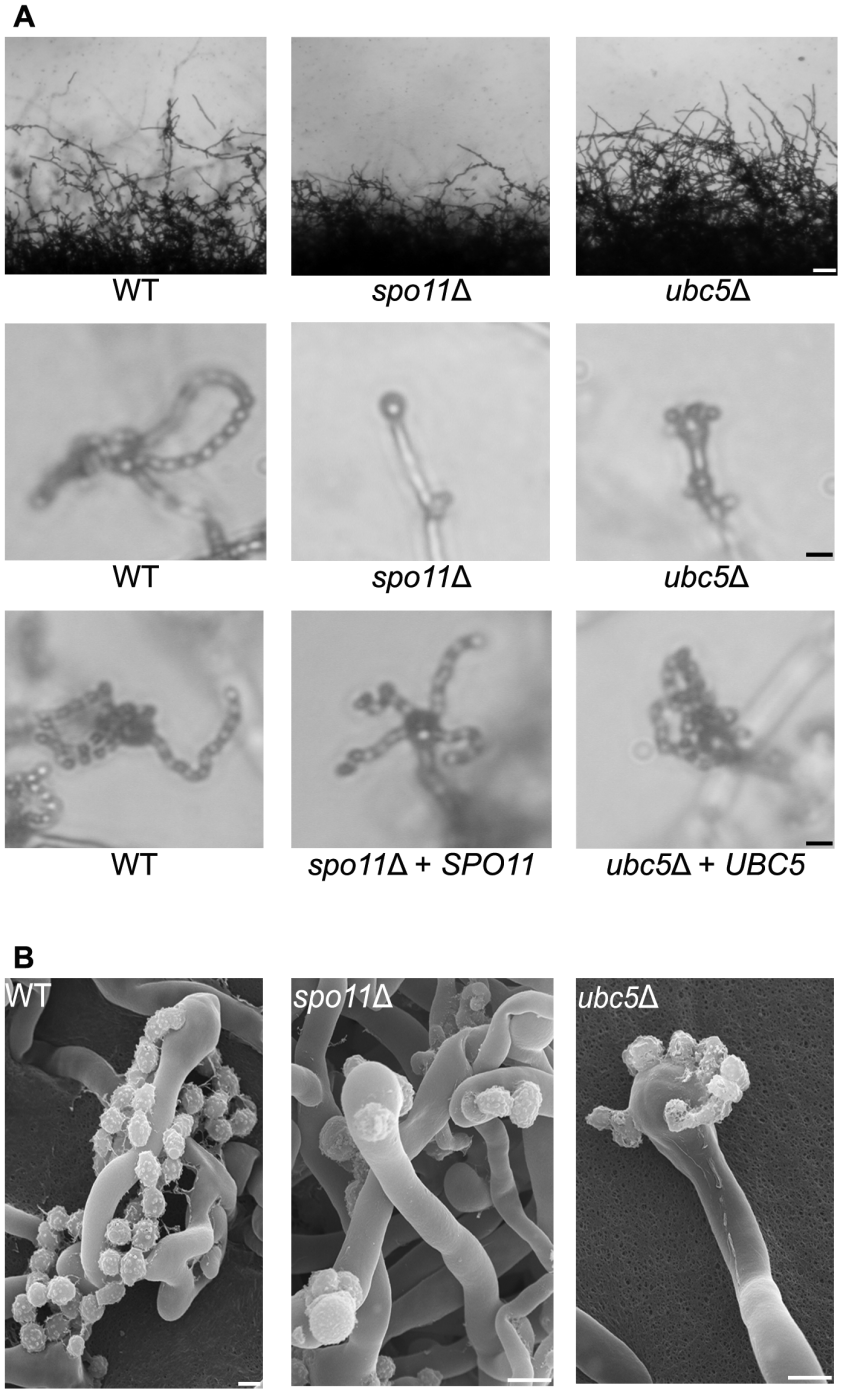
Spo11 and Ubc5 are required to complete sporulation during unisexual reproduction. Wild type XL280α and *spo11*Δ and *ubc5*Δ mutants were incubated on V8 medium in the dark at room temperature for 14 days. (**A**) Hyphal development was evaluated by light microscopy. The edges of the colony produced abundant hyphae in WT, *spo11*Δ, and *ubc5*Δ mutants (upper row). In XL280α, basidia were decorated with four spore chains, whereas in *spo11*Δ and *ubc5*Δ mutants, spore production was severely impaired and naked basidia or at most one or two short spore chains were observed. The scale bars represent 100 µm for the upper row and 10 µm for the middle rows. (**B**) Scanning electron microscopic analysis of sporulation defects during unisexual reproduction. The scale bars represent 5 µm.

Pheromone production and sensing initiate sexual development by inducing cell fusion and hyphal development. At the termini of the hyphae, a bulb-like structure known as the basidium forms where meiosis and sporulation occur. Previous studies in model organisms have shown that the endonuclease Spo11 and members of the ubiquitin complex govern meiosis and sporulation. In *S. cerevisiae* homozygous *spo11*/*spo11* mutants exhibit defects in meiosis and sporulation, while Ubc5 has been implicated in sporulation [33], [47], [48]. To establish the roles of Spo11 and Ubc5 in sporulation or meiosis in *C. neoformans*, both genes were deleted in XL280α via biolistic transformation and homologous recombination and two independent mutants were isolated for each. Unisexually reproducing cultures were incubated on filamentation-inducing media in the dark at room temperature for 10 days or longer. Hyphal development of the *spo11*Δ mutant was similar to wild type XL280α with hyphae covering the entire periphery of the colony (Figure 5A). Filamentation of the *ubc5*Δ mutants was also similar to *spo11*Δ and XL280α, albeit slower and slightly reduced. Deletion of either the *SPO11* or *UBC5* gene blocked sporulation during unisexual reproduction. In wild type XL280α, the majority of the basidia formed at the termini of the hyphae and were decorated with four long chains of basidiospores (Figure 5A). In the *spo11*Δ and *ubc5*Δ mutants, the basidia were either “bald” and completely lacked spores or had at most one or two spores and occasionally one very short chain of spores (Figure 5B). Complementation of *spo11*Δ and *ubc5*Δ mutants with *SPO11* and *UBC5* genes under the control of their native promoter and terminator, respectively, restored wild type sporulation.

**Figure 6:**
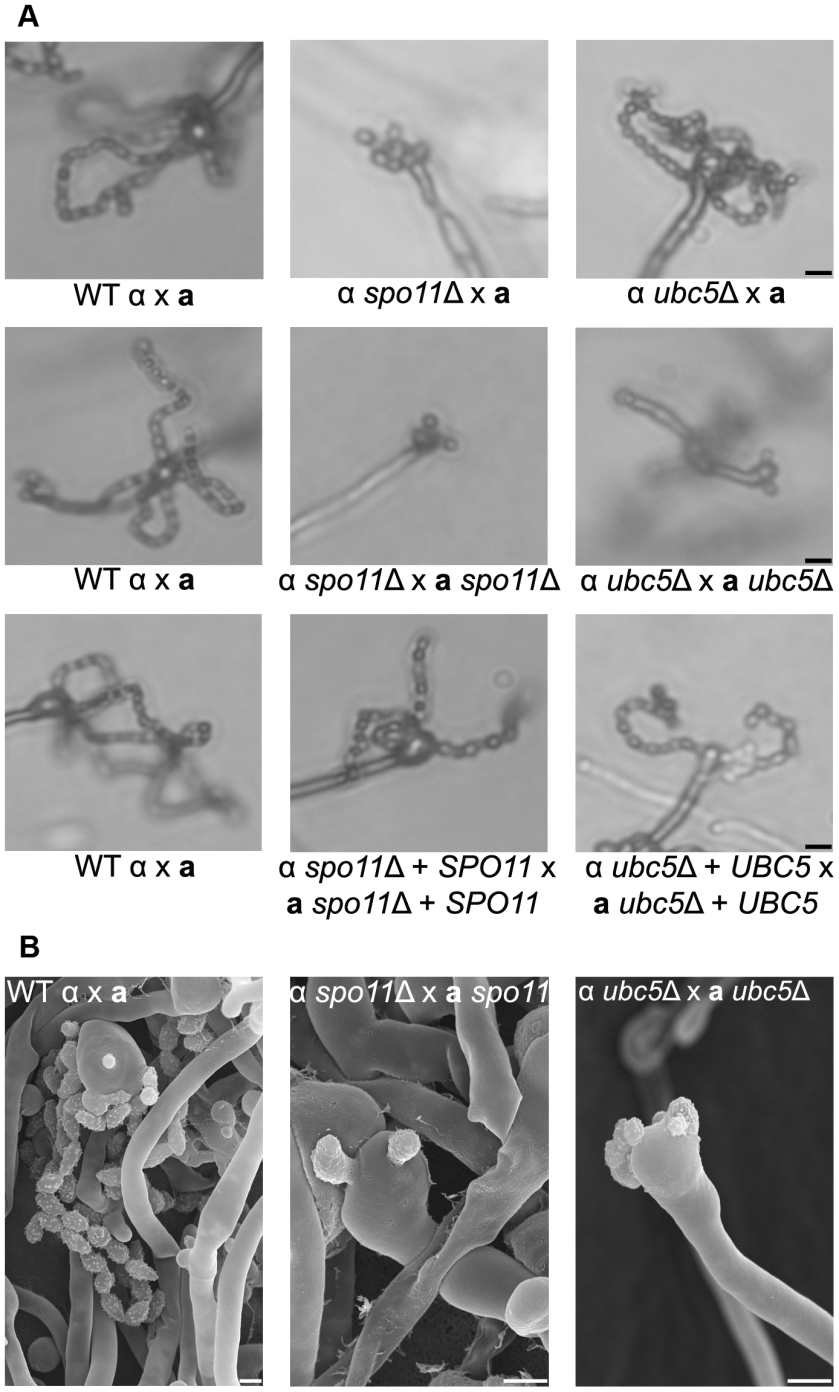
Spo11 and Ubc5 are necessary for sporulation during bisexual reproduction. Sporulation was assessed in wild type (XL280α×JEC20**a**), unilateral (α *spo11*Δ×JEC20**a**, α *ubc5*Δ×JEC20**a**), and bilateral (α *spo11*Δ×**a** *spo11*Δ, α *ubc5*Δ×**a** *ubc5*Δ) mating assays on V8 medium at room temperature in the dark for 7 days. (**A**) Spore production was evaluated by light microscopy. In WT crosses basidia produce abundant spores organized in four chains. In unilateral mutant matings spore production was similar to WT although modestly diminished. Bilateral mutants exhibited a severe defect in sporulation with none, one, or two spores. The scale bar represents 10 µm. (**B**) Scanning electron microscopic analysis of sporulation defects during bisexual reproduction. The scale bar represents 5 µm.

Bisexual reproduction results in significantly longer basidiospore chains in a shorter incubation time compared to unisexual reproduction. We deleted the *SPO11* and *UBC5* genes in the congenic mating partners JEC21α and JEC20**a** to assess their role in unilateral and bilateral crosses. In wild type matings (JEC21α×JEC20**a** and XL280α×JEC20**a**), the basidia formed four long spore chains on filamentation-inducing media after 7 days incubation in the dark at room temperature (Figure 6A). In unilateral crosses *spo11*Δ and *ubc5*Δ mutants were able to form four spore chains, but they were significantly shorter and in some cases, the basidia had only two or three short chains or even as few as only two or three spores. In bilateral *spo11*Δ×*spo11*Δ or *ubc5*Δ×*ubc5*Δ crosses, both mutants exhibited a severe sporulation defect. Most of the basidia were bald or were decorated with only one or two spores on the hyphae of the entire periphery (Figure 6B). Upon prolonged incubation (∼3 weeks), hyphal development increased with longer and more dense hyphae; however, the basidia failed to yield more than at most two or three spores (Figure 6 and Figure S7). This indicates that Spo11 and Ubc5 are critical for sporulation. A similar phenotype has been observed in the absence of the meiotic-specific recombinase Dmc1 in *C. neoformans* [18]. Deletion of *DMC1* results in basidia with a severe sporulation defect during bisexual and unisexual reproduction. In *S. cerevisiae*, Dmc1 is responsible for the repair of DNA double-strand breaks and homologous pairing during meiotic recombination [49]. Considering these results together, and based on the fact that *SPO11* is highly conserved, we hypothesize that its requirement for sporulation is attributable to roles in meiotic recombination occurring during both bisexual and unisexual reproduction.

### Spo11 is required for meiosis

An essential step prior to meiosis and sporulation is karyogamy. Karyogamy is characterized by two important steps: nuclear congression and nuclear fusion [50]. A series of genes are known to govern these steps in *S. cerevisiae*. In *C. neoformans*, how karyogamy is controlled remains largely unknown. A recent study identified a karyogamy gene, *KAR7*, which regulates nuclear fusion during bisexual and unisexual development [37]. Deletion of *KAR7* blocks nuclear fusion and severely impairs sporulation, similar to *spo11*Δ and *ubc5*Δ. To determine possible roles of Spo11 and Ubc5 in karyogamy, bisexual and unisexual reproduction patches of wild type and mutant partners were incubated for 10 days on V8 medium in the dark. The sexual reproduction cultures were fixed in 4% paraformaldehyde and nuclear positioning was visualized with Hoechst staining by fluorescence microscopy. Nuclear fusion in *spo11*Δ and *ubc5*Δ was normal in both hyphae and basidia and similar to wild type (Figure S6), suggesting that the *spo11*Δ and *ubc5*Δ sporulation phenotype is unrelated to nuclear fusion and that it is caused by defects in a step subsequent to karyogamy.

During meiosis, recombination plays a crucial role in promoting pairing of homologous chromosomes in yeast and mammals. Meiotic recombination is initiated by the formation of DNA double strand breaks (DSBs) induced by Spo11. Spo11 is highly conserved in eukaryotic species and mutations can lead to sterility or offspring with an abnormal chromosome number. Fertility in mice is abolished in the absence of Spo11 [51]; in *S. cerevisiae*, the survival rate of *spo11*Δ meiotic progeny is extremely low with high levels of aneuploidy [52], [53]. To determine the survival rates in *C. neoformans*, unisexual reproduction cultures of wild type XL280α and *spo11*Δ and *ubc5*Δ mutants were incubated on filamentation-inducing V8 medium in the dark at room temperature for 10 days. Using a microscope with a microdissection needle, we dissected spores from four different mating unisexual development assays for each strain. The results are summarized in Table 3. For wild type XL280α, 30 spores were dissected from each assay. The germination rate of dissected spores ranged from 40% to 70%, as previously observed. Due to sporulation defects, *spo11*Δ and *ubc5*Δ generated only one or two spores per basidium, and thus the number of dissected spores was lower than wild type. However, the germination frequency of dissected spores for both *spo11*Δ and *ubc5*Δ was significantly lower than the wild type and ranged from 0% to 9%. Extremely low viability of the unisexual progeny is usually attributed to chromosomal abnormalities, which is indicative of abnormal meiosis. Thus the low germination frequency of *spo11*Δ and *ubc5*Δ could be due to high levels of aneuploidy in the spores.

**Table 3:** Viability of wild type, *spo11*Δ, and *ubc5*Δ unisexual reproduction progeny.

Wild-type		*spo11*Δ		*ubc5*Δ	
# spores dissected	# spores germinated (%)	# spores dissected	# spores germinated (%)	# spores dissected	# spores germinated (%)
30	21 (70%)	22	2 (9%)	24	2 (8%)
30	12 (40%)	18	0 (0%)	17	1 (6%)
30	19 (63%)	25	1 (5%)	21	1 (6%)
30	16 (53%)	16	0 (0%)	16	0 (0%)

Based on sequence similarity, high homology, and the low germination rate of *spo11*Δ progeny, we reasoned that Spo11 may be required to catalyze DNA DSBs during meiosis in *C. neoformans*. Ionizing radiation has been found to suppress the meiotic defects of *spo11*Δ mutants and partially restore the wild type phenotype in *S. cerevisiae* and *C. cinerea* [54], [55]. To determine the role of Spo11 in DSBs during unisexual reproduction, cultures of XL280α, *ubc5*Δ, and *spo11*Δ were spotted on V8 medium to induce unisexual reproduction, incubated in the dark for 7 days, irradiated using X-rays at different doses (0, 1, 3, 5, 10, and 20 krad), and incubated for two additional days. Following irradiation, the *spo11*Δ mutant exhibited higher sporulation efficiency and a higher germination rate than the non-irradiated *spo11*Δ culture (Table 4 and Figure S8). A similar result was observed during *spo11*Δ×*spo11*Δ bisexual reproduction. Unlike the non-irradiated cultures of the *spo11*Δ bilateral cross (α *spo11*Δ×*spo11*Δ **a**), which produced very few spores, irradiated *spo11*Δ basidia had significantly more spores with considerably enhanced viability (Table 4 and Figure S8). Sporulation during both sexual cycles of WT and *ubc5*Δ was not significantly affected by irradiation, although viability was slightly lower, possibly due to higher rates of mutation (Table 4). XL280α, JEC21α, *ubc5*Δ, and *spo11*Δ mutants did not exhibit a growth defect in response to X-irradiation, and vegetative survival was similar between the WT and the mutants.

**Table 4:** X-irradiation-induced DSBs partially rescue the spore survival defect of *spo11*Δ mutants during bisexual and unisexual reproduction.

X-ray dose (kRad)	Viable cells/ml^a^	Unisexual reproduction progeny viability			Bisexual reproduction progeny viability			Total progeny
		XL280α (%)	*spo11*Δ (%)	*ubc5*Δ (%)	JEC21α×JEC20a (%)	α *spo11*Δ×*spo11*Δ a (%)	α *ubc5*Δ×*ubc5*Δ a (%)	
0	5.3×10^8^	23 (76%)	1 (3%)	2 (6%)	25 (83%)	2 (6%)	4 (13%)	30
1	5.4×10^8^	20 (66%)	0 (0%)	1 (3%)	28 (93%)	3 (10%)	3 (10%)	30
3	5.1×10^8^	21 (70%)	0 (0%)	3 (10%)	26 (86%)	0 (0%)	4 (13%)	30
5	4.8×10^8^	22 (73%)	2 (6%)	2 (6%)	24 (80%)	1 (3%)	5 (16%)	30
10	4.4×10^8^	18 (60%)	4 (13%)	0 (0%)	22 (73%)	5 (16%)	2 (6%)	30
20	3.1×10^8^	16 (53%)	7 (23%)	2 (6%)	20 (66%)	8 (26%)	3 (10%)	30

Cultures were incubated on V8 medium for six days for unisexual reproduction and four days for bisexual reproduction. Plates were irradiated at the designated dose and incubated in the dark for two days to allow spore production. A total of 30 spores were isolated from each culture.

a Viable CFU determined from vegetative growth on solid media independently for XL280α, *spo11*Δ, *ubc5*Δ, and JEC21α. The viable CFU for each strain at the same dose was similar with minor differences. Here we present the viability of XL280α as reference.

Taking these results together, we can conclude that 1) Spo11 is required for meiosis, likely by inducing DNA DSBs that promote recombination homologous pairing, and 2) Ubc5 plays an important role in sporulation that warrants further investigation. That Spo11 operates during unisexual reproduction provides further definitive evidence that this is a meiotic process.

## Discussion

We employed a classical genetic approach to identify genes that orchestrate unisexual reproduction in *C. neoformans*. The screen identified a number of genes that may be directly or indirectly implicated in the dimorphic switch during mating. Importantly, we successfully isolated five previously identified regulators, *CRG1*, *STE7*, *MAT2*, *ZNF2*, and *BWC2*, validating this approach. Our findings provide evidence to implicate *ZNF3*, *SPO11*, and *UBC5* in the control of hyphal development, meiosis, or sporulation during sexual differentiation. The severe defect in filamentation of the *znf3*Δ mutant provides evidence that Znf3 is a regulator of hyphal development during unisexual reproduction. The *znf3*Δ mutant phenotype is similar to that of *ste7*Δ and *mat2*Δ mutants and of other components of the pheromone pathway. In addition, RT-PCR analysis showed that the expression profiles of these mutants and *znf3*Δ followed a similar pattern during bisexual and unisexual reproduction. Hyphal development during bisexual reproduction in the *znf3*Δ mutant is also affected; however, the defect is less pronounced, and filamentation is mainly delayed rather than abolished. These defects are less severe than the defects observed in *ste7*Δ, *mat2*Δ, or *znf2*Δ α-**a** bilateral mutant sexual reproduction assays.

**Figure 7:**
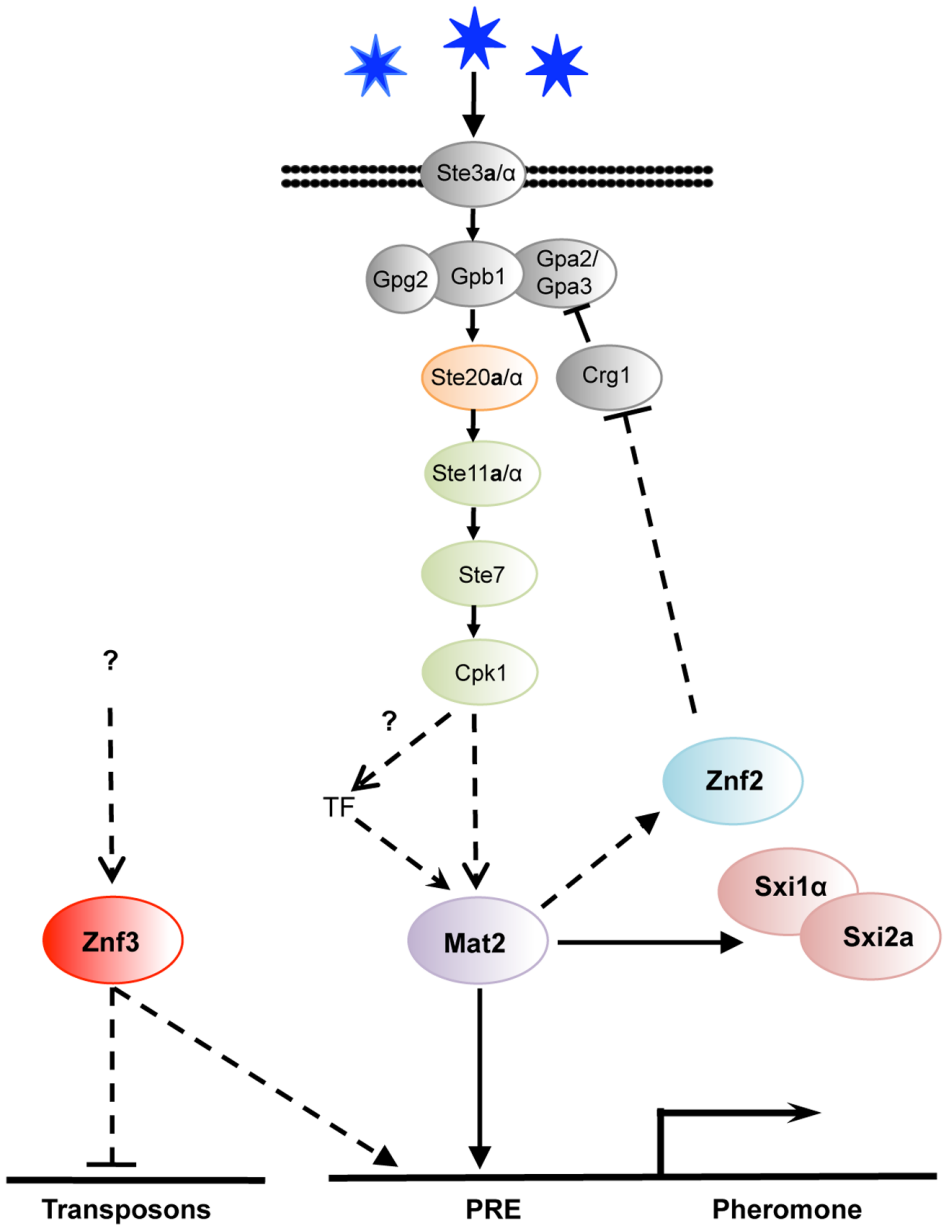
Znf3 regulates pheromone expression activating the pheromone signaling cascade and promotes transposon silencing. Mat2 is the transcription factor target of the pheromone signaling cascade that binds to the pheromone response element (PRE) in the promoter of the pheromone gene. Znf3 is required for induced levels of the pheromone and transposon silencing. Znf3 may bind additional *cis*-regulatory element to inhibit transposon mobilization during sexual development or function by binding to RNA targets.

Regulation of pheromone expression is a key function of the components of the MAPK signaling cascade. Pheromone expression is dramatically attenuated in *ste7*Δ and *mat2*Δ mutants, as shown here and in previous studies [26]. A striking finding was the inhibition of *MAT2* and pheromone expression in the *znf3*Δ mutant. Interestingly, we found that *MAT2* transcription increases during mating (Figure S3), and *znf3*Δ and *ste7*Δ mutations have a similar impact on the transcriptional control of *MAT2* and *MF*α*1* during bisexual reproduction (Figure 3B), indicating that they may be in the same or related pathways. Epistasis analysis suggests Znf3 does not function upstream or downstream of Mat2. The lower expression of *MAT2* could be attributable to the absence of pheromone in the *ste7*Δ and *znf3*Δ mutants. Pheromones activate the pathway that leads to expression of targets, including *MAT2*, whose transcription is regulated by itself, an unknown factor, or both. Based on these findings, we hypothesize that Znf3 imposes a mode of regulation on Mat2 via direct or indirect control of the pheromone through an independent signaling cascade that remains to be defined (Figure 7).

We found that *ZNF3* transcription levels are similar during vegetative and hyphal growth, and *ZNF3* may require activation through phosphorylation by the MAPK signaling cascade or via a different pathway. A recent study by Kruzel *et al.* showed that Mat2 is the pheromone response transcription factor, with a consensus binding motif present as the pheromone response element (PRE) in key mating genes [27]. The defined PRE sequence lies upstream of multiple genes (i.e. *MF*α*1*, *MF*α*2*, *MF*α*3*, *STE3*α, *STE12*α, *SXI1*α, and *RAM1*) and is recognized specifically by Mat2 [27]. However, extensive study of the MAPK signaling cascade in *S. cerevisiae* revealed that the promoter regions of several PRE-containing genes bear additional *cis*-regulatory elements that are recognized by different transcription factors or other elements, conferring additional levels of control [56], [57], [58]. Different environmental cues may govern these additional transcription factors, likely through pheromone-independent signaling cascades. It is possible that similar networks may contribute to PRE activation in *C. neoformans*. Znf3 may regulate the pheromone genes through another cis-regulatory element induced by a different environmental cue or an independent pathway that confers developmental specificity (Figure 7).

The identification and characterization of the targets of this novel factor will be essential to elucidate its molecular functions during mating. Surprisingly, deletion of *ZNF3* increased the expression of transposons during unisexual reproduction. Combined with the fact that a similar transcriptional profile is observed in RNAi mutant strains, this leads to the conclusion that Znf3 may play a role in SIS and could be required to defend the genome against transposon mobilization. It is of interest that this protein appears to play a dualistic role during sex. It acts as an activator of pheromone expression and, simultaneously, as an inhibitor of transposon transcription (Figure 7). A possible explanation is that Znf3 plays a pivotal role in transposon silencing and is a molecular link between SIS and sexual development. Future studies will focus on the mechanism of transposon silencing by Znf3 and explore the links with sexual development.

Sexual reproduction has several stages that are each precisely regulated. Meiosis and sporulation are the ultimate steps. Our screen revealed two genes implicated in meiosis and/or sporulation. The first, *UBC5*, has a homolog in *S. cerevisiae* that is involved in ubiquitin-targeted degradation [33]. The second, *SPO11*, is considered a hallmark meiotic gene [59]. We found that deletion of either gene in *C. neoformans* results in barren basidia or basidia with only one or two spores with a very low germination rate. Deletion of *DMC1*, a previously identified meiosis-specific factor, also results in a severe sporulation defect, similar to the *spo11*Δ mutant [18]. We found that X-irradiation-induced DSBs partially restored the WT phenotype and enhanced the germination rate of the *spo11*Δ progeny. Along with the observation that Spo11 shares homology with the *S. cerevisiae* ortholog, we suggest that *Cryptococcus* Spo11 is required for initiating meiosis by catalyzing DSBs and inducing meiotic homologous recombination during bisexual and unisexual development.

Deletion of *UBC5* shares a similar phenotype with the *spo11*Δ mutant, namely a severe sporulation defect and largely inviable meiotic progeny. This result is consistent with presumptive roles in meiosis or sporulation. In addition, *Cryptococcus* Ubc5 exhibits significant homology and identity throughout the fungal kingdom, possibly reflecting a conserved role. The *S. cerevisiae* Ubc5 homolog has been implicated in sporulation; however, the precise role(s) remains unknown. Ubiquitin-conjugating enzymes are associated with structurally similar but distinct recognition subunits that direct these enzymes to proteins that are targeted for degradation. Ubc5 is associated with the proteasome in *S. cerevisiae*, along with multiple E2 enzymes, and there are likely to be multiple physiological substrates, only some of which are involved in sporulation. Based on the highly conserved ubiquitin-conjugating enzyme E2 catalytic domain in *Cryptococcus*, we hypothesize that the role of Ubc5 involves degradation of multiple as yet unknown targets, and depletion of some of these targets is required for meiotic entry or sporulation.

Hallmarks of sexually reproductive species are the presence of recombinant progeny in the population and a group of highly conserved meiotic genes termed the “meiotic toolkit” in their genomes [59]. These genes represent the best markers for the capacity for a complete sexual cycle in a fungal species. Meiosis toolkit genes are present in *C. neoformans* and both our study and others have shown that two of these, *SPO11* and *DMC1*, are required for meiosis and sporulation during bisexual and unisexual reproduction [18]. However the majority of meiotic gene candidates remain uncharacterized. *S. cerevisiae* represents an extensively studied meiotic system. Hop1 secures the synapses between homologous chromosome by binding DNA at sites where DSB will be induced by the Spo11 endonuclease [32], [60]. Dmc1 is required for DNA DSBs repair and catalyzes strand invasion and the formation of Holiday junctions that are stabilized by the Mnd1/Hop2 complex [61], [62]. Finally, Msh4 forms a dimer with Msh5 and facilitates crossing over, while Rec8 mediates cohesion between sister chromatids [63], [64]. Although the sequence of meiotic events is highly conserved in sexual species, the system exhibits exceptional plasticity in the fungal kingdom. For example, no true meiosis has been observed in *C. albicans*, which has a parasexual cycle, where random, concerted, chromosome loss occurs following mating, despite the presence of many meiosis-specific genes [65]. On the other hand, sporulation in *C. lusitaniae* is relatively efficient and involves Spo11-induced meiotic recombination, although it is missing multiple meiotic genes [66]. Moreover, in *U. maydis*, a basidiomycete, sexual development is efficient; however, it lacks key regulators of meiosis (Dmc1, Hop1, Hop2, and Mnd1) and has incorporated novel factors like homologs of *H. sapiens BRCA2* and *DSS1* [67]. In C. *neoformans*, we have found that Spo11 is required for meiosis, evidently for the induction of DSBs; however, taking into account the mechanistic and evolutionary plasticity of meiotic events in fungal species, the survey of the genome for the repertoire of meiotic-specific factors alone may well miss elements that require genetic approaches such as those applied here to unveil. Future research should focus on the genetic and evolutionary analysis of these and other unidentified genes to explore a possible conserved mechanism or a diverse rewiring network that controls meiosis and sporulation during *C. neoformans* unisexual reproduction.

## Materials and Methods

### Strains, media, and growth conditions

*C. neoformans* strains and plasmids used in this study are summarized in Table S3. *Cryptococcus* strains were maintained in −80°C glycerol stocks and grown at 30°C on rich YPD media (Yeast extract Peptone Dextrose). Strains harboring dominant selectable markers were grown on YPD containing nourseothricin (NAT) or G418 (NEO). Mating assays were performed on 5% V8 juice agar medium (pH = 7) in the dark at room temperature for the designated incubation period. To visualize spores and spore chains, mating assays were conducted on either V8 solid agar or on MS (Murashige and Skoog) medium minus sucrose (Sigma-Aldrich).

### Insertional mutagenesis and phenotypic analysis of the mutants

*Agrobacterium tumefaciens*-mediated transformation was performed as previously described [29]. *A. tumefaciens* strain EHA105 transformed with the pPZP-NAT1cc plasmid was grown on Luria-Bertani (LB) medium containing kanamycin for 48 h at 25°C with shaking. The cells were washed briefly with sterile ddH_2_O and diluted in induction medium containing 100 µM acetosyringone to obtain an optical density of 0.15 at 660 nm. The cells were then incubated for 6 h while shaking at room temperature. The *C. neoformans* serotype D strain XL280α was grown overnight in YPD, washed with induction medium, and resuspended to a final density of 10^7^ cells/ml. Equal aliquots of *C. neoformans* and *A. tumefaciens* (200 µl) were mixed and plated on induction medium agar containing acetosyringone. The cells were co-cultured for three days, scraped from the plates, and transferred to selective medium (YPD+NAT+100 µg/ml cefotaxime). The colonies were transferred into 96-well plates and grown in YPD+NAT+100 µg/ml cefotaxime as shaking cultures. A total of 6,100 transformants were spotted on V8 solid medium, incubated for 14 days in the dark at room temperature, and examined for hyphal development using light microscopy. Mutants with a stable filamentation defect were selected and clustered into groups based on similar phenotypes. Linkage between NAT resistance and the phenotype was assessed by crossing the insertional mutants with the JEC20**a** mating partner. Individual spores were isolated using a microdissection microscope, and the progeny were screened for the mutant phenotypes and NAT resistance. To identify the insertion sites, genomic DNA was digested with multiple restriction enzymes, purified, self-ligated, and subjected to inverse PCR using the pre-selected primers AI076/AI077. The PCR products were sequenced and the flanking regions were used in BLAST searches against the *Cryptococcus* genome to identify the mutated genetic loci [68].

### Genomic DNA preparation

Cells were grown overnight in 50 ml YPD at 30°C in shaking cultures. The cells were harvested, washed three times with ddH_2_O, and frozen at −80°C overnight. The next day, they were lyophilized overnight and either stored at −20°C or genomic DNA was isolated using the CTAB protocol as previously described [69].

### Gene disruption, complementation, and overexpression

Genes of interest were disrupted using an overlap PCR approach [14], [70]. The 5′ and 3′ flanking sequences of *ZNF3*, *SPO11*, and *UBC5* were amplified from either XL280α or JEC21α genomic DNA. The dominant selectable markers *NAT* and *NEO* were amplified from plasmids pAI3 and pJAF1 respectively, with the universal primers M13F and M13R. The flanking sequences and the selectable markers were used to generate full-length deletion cassettes in an overlap PCR reaction. The construct was purified, precipitated onto gold microcarrier beads (0.6 mm, Bio-Rad), and introduced into strains XL280α or JEC20**a** via biolistic transformation as previously described [71]. Genes were replaced via homologous recombination and confirmed with PCR and Southern hybridization. Primers used to generate mutations are listed in Table S4. For complementation, the wild type *SPO11* and *UBC5* genes, along with their endogenous promoters and terminators, were amplified from XL280α genomic DNA with primer pairs JOHE37665/JOHE37666 and JOEH37667/JOHE37668, respectively, and cloned into the pJAF12 and pJAF13 vectors [14]. The plasmids were confirmed by sequenced, and *spo11*Δ and *ubc5*Δ mutants were biolistically transformed. The transformants were screened for spore production during mating by light microscopy. The wild type *ZNF3* gene is exceptionally large (∼7,000 bp including promoter and terminator) and the construction of a complementation allele was challenging. To show that the hyphal defect was attributable to disruption of the gene, we screened and found a consistent afilamentous phenotype in several insertional mutants with independent insertions in *ZNF3* and also in two independently derived *znf3*Δ mutants. To generate the *ZNF3* overexpression plasmid pMF3, the wild type *ZNF3* allele was amplified from XL280α genomic DNA using primer pair JOHE21470/JOHE21471. The gene was cloned into the pXL1 vector under the control of the constitutively expressed *GPD1* promoter [72]. *Cryptococcus* strains were transformed with the circular plasmid via biolistic transformation. Independent transformants were selected for RNA extraction and screened to determine the expression levels of *ZNF3* by RT-PCR.

### RNA extraction and RT-PCR

RNA from cells undergoing bisexual reproduction was isolated by mixing equal amounts of **a** and α cells in an eppendorf tube. Then, 5 ml of cells were spotted on V8 medium and incubated for 24 hours at room temperature in the dark. RNA was isolated from unisexual reproduction cultures by spotting 5 ml of α cells on V8 for 48 hours at room temperature in the dark. Mating cultures were harvested, washed with ddH_2_O, and lyophilized overnight. RNA was extracted using TRIzol Reagent following the manufacturer's instructions (Invitrogen). Then, 5 µg of total RNA were subjected to DNAse treatment using Turbo DNAse (Ambion), and single-stranded cDNA was synthesized by AffinityScript RT-RNAse (Stratagene). Quantitative Real-Time PCR (RT-PCR) was performed on an Applied Biosystems 7500 Real-Time PCR System using Brilliant SYBR Green qRT-PCR master mix (Stratagene). For each target, a “no template control” was included and melting curves were analyzed to exclude primer artifacts and a single PCR product. All assays were conducted in triplicate. Data was normalized relative to the reference gene *GPD1*, and expression was determined using the 2^−ΔΔCT^ approach. The primers used for RT-PCR are summarized in Table S4. The Student's t-test was employed to determine if the relative expression of the mutants was significantly different compared with the wild type (significance p<0.05).

### Mating assays and microscopy

Each **a** and α mating partner was grown on YPD solid agar overnight at 30°C. The cells of each mating type were either mixed for bisexual reproduction or spotted individually for unisexual reproduction on V8 solid medium (pH = 7) for 7 or 14 days at room temperature in the dark. Hyphal structures and spores were visualized with an Eclipse E400 microscope (Nikon) equipped with a DXM1200F digital camera (Nikon) and interfaced with ACT-1 software (Nikon). Individual spores were isolated using a microdissection microscope equipped with a 25-µm microneedle (Cora Styles Needles ‘N Blocks, Dissection Needle Kit) as previously described [73].

To visualize nuclear position and fusion in basidia and the hyphae, mating assays were fixed in 4% paraformaldehyde for 20 minutes and then washed three times with sterile PBS. The cells were stained with 1 mg/ml Hoechst dye, incubated for 20 min, and washed with sterile PBS. The mating structures were mounted and captured by brightfield, differential interference (DIC), and fluorescence microscopy (DAPI channel) using a Zeiss Axioskop 2 PLUS equipped with an AxioCam MRm camera (Carl Zeiss Inc., Thornwood, NY).

For scanning electron microscopy, the mating samples were processed at the North Carolina State University Center for Electron Microscopy, Raleigh, NC, USA. Briefly, 1 mm^3^ blocks of matings performed on MS medium were excised from the agar and fixed with 0.1 M Na cacodylate buffer (pH = 6.8) containing 3% glutaraldehyde for several weeks at 4°C. The block was then rinsed with cold 0.1 M Na cacodylate buffer (pH = 6.8) three times and post-fixed in 2% osmium tetroxide in cold 0.1 M Na cacodylate buffer (pH = 6.8) for 2.5 hours at 4°C. Before viewing, the samples were critical-point dried with liquid CO_2_ and sputter coated with 50 Å of gold/palladium using a Hummer 6.2 sputter coater (Anatech). The samples were viewed at 15 kV with a JSM 5900LV scanning electron microscope (JEOL) and captured with a Digital Scan Generator (JEOL) image acquisition system. All of the microscopic images were processed with PhotoShop (Adobe).

### Cell-cell fusion assay

To determine the cell-cell fusion frequency of wild type and mutants cells during bisexual reproduction, wild type strains XL561 (XL280α *NAT*) and XL491 (JEC20**a** *NEO*) and *znf3*Δ mutants MF38 (XL280α *znf3*Δ::*NAT*) and MF40 (JEC20**a** *znf3*Δ::*NEO*) were grown overnight in YPD liquid medium at 30°C. The cells were washed twice with ddH_2_O and diluted to a final density of 10^7^ cells/ml. Both unilateral and bilateral mutant matings were assessed using XL926α (XL280α *mat2*Δ::*NAT*) and XL961**a** (JEC20**a** *mat2*Δ::*NEO*) mutants as negative controls. Equal amounts of cells were mixed (500 µl) and spotted on V8 medium (pH = 7) and incubated for 15 and 24 hours in the dark at room temperature. The cells were then removed, washed with ddH_2_O, and plated in serial dilution on YPD+NEO+NAT media to select for cell-cell fusion products. The cells were incubated for five days at room temperature. Cell-cell fusion efficiency was measured by counting the average number of double drug resistance CFU/total CFU in the wild type and mutant crosses.

### Microarray and data analysis

To obtain RNA for microarray analysis, XL280α and the congenic *znf3*Δ mutant were grown in liquid cultures, washed in water, and spotted on V8 medium for 24 hrs. The unisexual reproduction cultures were harvested, washed in water, and lyophilized overnight. Three independent cultures were isolated for each strain and prepared for RNA isolation as biological replicates. The RNA was isolated using the RiboPure™-Yeast Kit (Ambion) according to the manufacturer's instructions (Life Technologies #AM1926). cDNA was synthesized using AffinityScript reverse transcriptase (Stratagene). cDNA was Cy3/Cy5-labeled and hybridized to a *C. neoformans* 70-mer microarray slide developed by the *Cryptococcus* Community Microarray Consortium (Washington University, St. Louis, MO). The arrays were washed and scanned with a GenePix 4000B scanner (Axon Instruments at Duke University DNA Microarray Core Facility) [74]. Three independent DNA microarrays were performed with three independent biological replicates. For statistical analysis, data was analyzed using GeneSpring software (Agilent) by employing Lowess normalization, reliable gene filtering, ANOVA analysis (significance p<0.05), and Microsoft Excel Software.

For the hierarchical clustering analysis the microarray expression data was carried out using the Limma Bioconductor package of the R statistical programming language [75], [76]. Briefly, normal exponential background subtraction was applied to the data followed by Lowess normalization. Significance of differential expression for the comparisons of the wild type versus mutant strains was then carried out using an empirical Bayes' moderated t-test. The False Discovery Rate method was applied to correct for multiple hypothesis testing [77]. Hierarchical clustering (complete-linkage, correlation distance) was accomplished using the R statistical programming language.

### Fluorescence Activated Cell Sorting (FACS) analysis

Strains were processed for flow cytometry using XL280α as a haploid control and XL143 as a diploid control, as previously described [19]. Cells were grown overnight in YPD liquid media at 30°C, harvested, and washed with PBS buffer. They were fixed with 70% ethanol and incubated at 4°C overnight. The strains were washed with 1 ml of NS buffer [(10 mM Tris-HCl (pH = 7.2), 250 mM sucrose, 1 mM EDTA (pH = 8.0), 1 mM MgCl_2_, 0.1 mM CaCl_2_, and 0.1 mM ZnCl_2_], treated with 1 mg/ml RNase, and stained with 10 mg/ml propidium iodide in a final volume of 200 µl at 4°C overnight. Then, 50 µl of stained cells were diluted in 2 ml of 50 mM Tris-HCl (pH = 8.0) and sonicated for 1 min. Flow cytometry was performed on 10,000 cells and analyzed on the FL1 channel with a Becton-Dickinson FACScan at the Duke Cancer Institute Flow Cytometry Shared Resource.

### X-irradiation procedures

Strains were grown overnight in liquid cultures, washed with water, and spotted on V8 medium pH = 7. It is crucial to impose irradiation treatment soon after karyogamy is completed and after the initiation of premeiotic DNA synthesis; however, the timing of these two processes are unknown in *Cryptococcus*. Therefore, we X-irradiated cultured crosses undergoing sexual reproduction at different time points (3, 4, 5, 6, 7, and 8 days incubation on V8 media) at a dose rate of 420 rad/min in an X-RAD 320 X-ray irradiator set at 320 kilovolts and 10 milliamps. Unirradiated cultures were plated in the same fashion and transferred to the X-ray machine to avoid inadvertent effects of these manipulations. Vegetative asexual cultures were plated in serial dilutions on solid YPD media and irradiated together with the sexual cultures to determine the vegetative survival rate and the presence of growth defects in response to radiation. After X-irradiation, crossing cultures undergoing sexual reproduction were incubated at room temperature in the dark for two additional days and assessed for sporulation efficiency with light microscopy. Germination rate was determined by isolating individual spores micro-manipulation from each culture and incubating these on YPD media to determine the number that formed a colony.

## Supporting Information

Figure S1 ***Cryptococcus neoformans*** **sexual cycle.** (**A**) During bisexual reproduction opposite mating type cells secrete pheromones under nutrient limiting conditions, initiating the formation of conjugation tubes leading to cell-cell fusion. The two cells form a diploid heterokaryon, which initiates filamentous growth. At the apex of the filaments, specialized structures known as basidia form where nuclear fusion and meiosis occur. Multiple rounds of mitosis and budding produce chains of basidiospores. During unisexual reproduction α cells may undergo an early or late diploidization event to generate a diploid or haploid monokaryotic hyphae respectively. In both cases meiosis and sporulation produce meiotic progeny with long chains of basidiospores (Redrawn from Idnurm et al. [11]). (**B**) Early diploidization in α cells may be induced in ménage à trois matings where **a** cells donate pheromone and stimulate α-α cell fusion or in the absence of a compatible mating partner by cell-cell fusion between mother and daughter cells. Early diploidization may also occur through endoreplication where cells undergo DNA replication and transition from a haploid to diploid nucleus or they undergo nuclear division followed by nuclear re-fusion without cell division. (**C**) The cells budding from the haploid or diploid monokaryotic hyphae (blastospores) are indicative of the ploidy of the hyphae. Blastospores were isolated from different hyphae through microdissection. Blastospores from 5/19 (26%) and 9/24 (37.5%) hyphae were found to be diploid whereas blastospores from 14/19 (74%) and 14/24 (62.5%) hyphae were haploid according to FACS analysis. During unisexual reproduction hyphae development is more robust in diploid isolates compared to haploids, as previously observed.
(TIF)

Figure S2 **Protein organization of Znf3, Spo11, and Ubc5.** Znf3 is a large protein (1,561 amino acids) unique in the *Cryptococcus* genus. Due to the presence of three zinc finger domains and possible localization to the nucleus based on the presence of predicted nuclear localization signals (NLS), this protein may act as a transcription factor or could be involved in other nucleic acid (DNA or RNA)-regulated processes or trafficking. A coiled coil region could mediate interactions with an unknown co-factor or itself. Spo11 has a conserved DNA topoisomerase VI domain that mediates its role in generating DNA DSBs during meiosis. Ubc5 has a conserved ubiquitin-conjugating E2 enzyme domain that spans the entire protein. In the insertion mutants, the genes have been disrupted at the designated sites by the NAT drug resistance marker. Motif predictions were based on the genome of JEC21α and the motif scan tool PROSITE. Protein localization sites were identified using the WoLF PSORT program.
(TIF)

Figure S3 **Expression profiles of** ***ZNF3*** **,** ***MAT2*** **,** ***SXI1*** **α and** ***MF*** **α** ***1*** **during bisexual reproduction.** WT α and **a** cells were mixed in equal numbers, co-cultured on V8 medium, and incubated in the dark for 0 (vegetative growth), 24, 48, or 72 hrs. The cells were harvested and RNA was isolated from both yeast cells and hyphae. RT-PCR showed that *ZNF3* expression during bisexual reproduction remained similar to vegetative growth. *MAT2*, *SXI1*α, and *MF*α*1* expression increased significantly at 24 hrs. The error bars represent the standard deviations from the mean for the three biological replicates.
(TIF)

Figure S4 **Expression profile of genes encoding Cpk1, Crg1, and Crg2.** Cells were incubated for 24 hours on V8 medium for bisexual and 48 hours for unisexual reproduction. Yeast and hyphal cells were harvested and RNA was isolated. Expression was measured by RT-PCR in wild type, *ste7*Δ, *mat2*Δ, *znf2*Δ, *sxi1*αΔ, and *znf3*Δ mutants. (**A**) The MAP kinase *CPK1* transcriptional profile is similar to *MAT2*, and its expression is possibly regulated by the pheromone signaling cascade. (**B**) Expression of the negative regulator *CRG1* was significantly increased in the *znf2*Δ mutant. The elevated levels of *CRG1* may contribute to the severe filamentation defect of *znf2*Δ mutants during unisexual reproduction. *CRG2* expression in pheromone response mutants was similar to wild type (* indicates P<0.05 and ** indicates P<0.005 compared to the WT). The error bars represent the standard deviations from the mean for the three biological replicates.
(TIF)

Figure S5 **Microarray analysis of gene expression in** ***znf3*** **Δ,** ***mat2*** **Δ and XL280 during unisexual reproduction.** Microarray data was obtained from three independent experiments for each mutant and the wild type during unisexual reproduction. Hierarchical cluster analysis is presented in the heatmap as z-score normalized log_2_ expression values. The columns represent each microarray experiment whereas the rows represent the genes filtered by statistical significance (p<0.05). Green indicates an increase in expression level, red indicates a decrease, and black indicates that the expression level did not change between the different isolates.
(TIF)

Figure S6 **Localization of nuclei during unisexual reproduction.** Wild type, *spo11*Δ, and *ubc5*Δ mutants were incubated on MS medium in the dark at room temperature for 10 days. Small patches of agar were excised and stained with Hoechst dye. Nuclear positioning was visualized with fluorescent microscopy. In wild type unisexual reproduction hyphae are monokaryotic with distinct nuclei in each hyphal compartment. In *spo11*Δ and *ubc5*Δ mutants, the unisexual hyphae are similar to wild type. The *kar7*Δ karyogamy mutant impairs hyphal growth by preventing early nuclear diploidization, and also leads to a sporulation defect by blocking late nuclear diploidization, leading to paired unfused nuclei in the basidium [37]. The *spo11*Δ, and *ubc5*Δ mutants have only one nucleus in the basidium similar to the wild type and no hyphal growth impairment, consistent with no observed defects in karyogamy. The scale bar represents 10 µm.
(TIF)

Figure S7 **Scanning electron microscopic analysis of sporulation defects.** The upper panel shows wild type bisexual and unisexual reproduction leading to hyphae with basidia decorated with long spore chains that formed after 14 days incubation on MS media. The middle and lower panels depict sporulation defects during bisexual and unisexual reproduction of *spo11*Δ and *ubc5*Δ mutants, respectively. The scale bars represent 1 µm.
(TIF)

Figure S8 **X-irradiation partially suppresses the sporulation defect of** ***spo11*** **mutants.** Bisexual and unisexual reproduction cultures were incubated for 7 days on V8 medium, irradiated with the designated dose, and incubated 2 additional days in the dark at room temperature. The first row depicts the sporulation defect of *spo11*Δ and *ubc5*Δ mutants of unirradiated cultures. The second and third rows show that X-irradiation partially restored spore production in the *spo11*Δ mutant, while sporulation of the irradiated *ubc5*Δ mutant was similar to the unirradiated samples. The scale bar represents 10 µm.
(TIF)

Table S1 **A list of genes downregulated in the** ***znf3*** **Δ and** ***mat2*** **Δ mutants during bisexual and unisexual reproduction.**
(XLS)

Table S2 **A list of genes upregulated in the** ***znf3*** **Δ mutant during unisexual reproduction (p<0.05).**
(XLS)

Table S3 **Strains and plasmids used in this study.**
(DOC)

Table S4 **Primers used in this study.**
(DOC)

Text S1 **Genomic and protein organization of Znf3, Spo11, and Ubc5.**
(DOC)
