# Supplementary figures and images for: Genetic Circuits that Govern Bisexual and Unisexual Reproduction in Cryptococcus neoformans
Source: PLoS Genet. 2013 Aug 15;9(8):e1003688. doi: 10.1371/journal.pgen.1003688 (PMC3744442; doi:10.1371/journal.pgen.1003688)

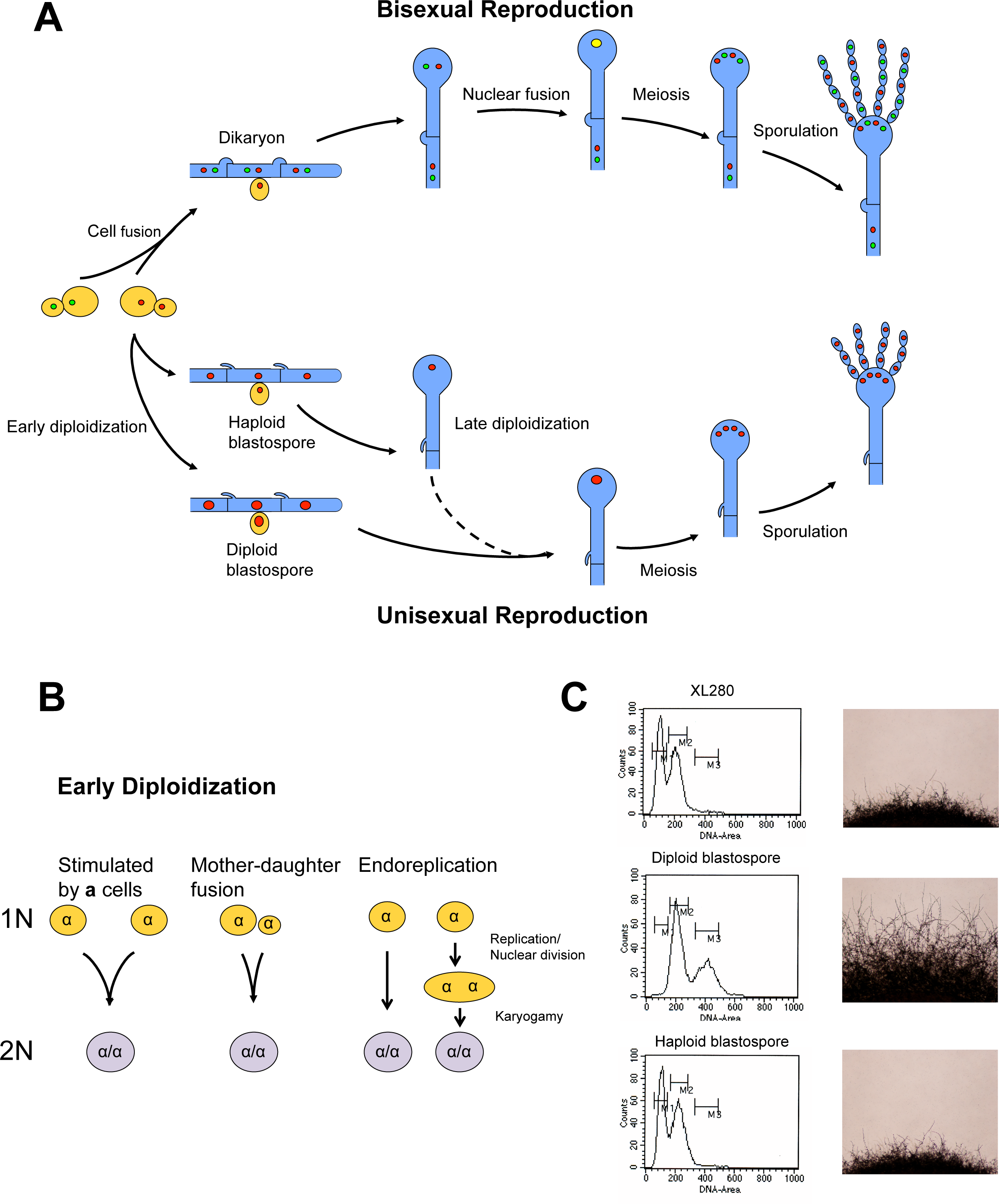

Supplement: Figure S1 — Cryptococcus neoformans sexual cycle. (A) During bisexual reproduction opposite mating type cells secrete pheromones under nutrient limiting conditions, initiating the formation of conjugation tubes leading to cell-cell fusion. The two cells form a diploid heterokaryon, which initiates filamentous growth. At the apex of the filaments, specialized structures known as basidia form where nuclear fusion and meiosis occur. Multiple rounds of mitosis and budding produce chains of basidiospores. During unisexual reproduction α cells may undergo an early or late diploidization event to generate a diploid or haploid monokaryotic hyphae respectively. In both cases meiosis and sporulation produce meiotic progeny with long chains of basidiospores (Redrawn from Idnurm et al. [11]). (B) Early diploidization in α cells may be induced in ménage à trois matings where a cells donate pheromone and stimulate α-α cell fusion or in the absence of a compatible mating partner by cell-cell fusion between mother and daughter cells. Early diploidization may also occur through endoreplication where cells undergo DNA replication and transition from a haploid to diploid nucleus or they undergo nuclear division followed by nuclear re-fusion without cell division. (C) The cells budding from the haploid or diploid monokaryotic hyphae (blastospores) are indicative of the ploidy of the hyphae. Blastospores were isolated from different hyphae through microdissection. Blastospores from 5/19 (26%) and 9/24 (37.5%) hyphae were found to be diploid whereas blastospores from 14/19 (74%) and 14/24 (62.5%) hyphae were haploid according to FACS analysis. During unisexual reproduction hyphae development is more robust in diploid isolates compared to haploids, as previously observed. (TIF) [file pgen.1003688.s001.tif]

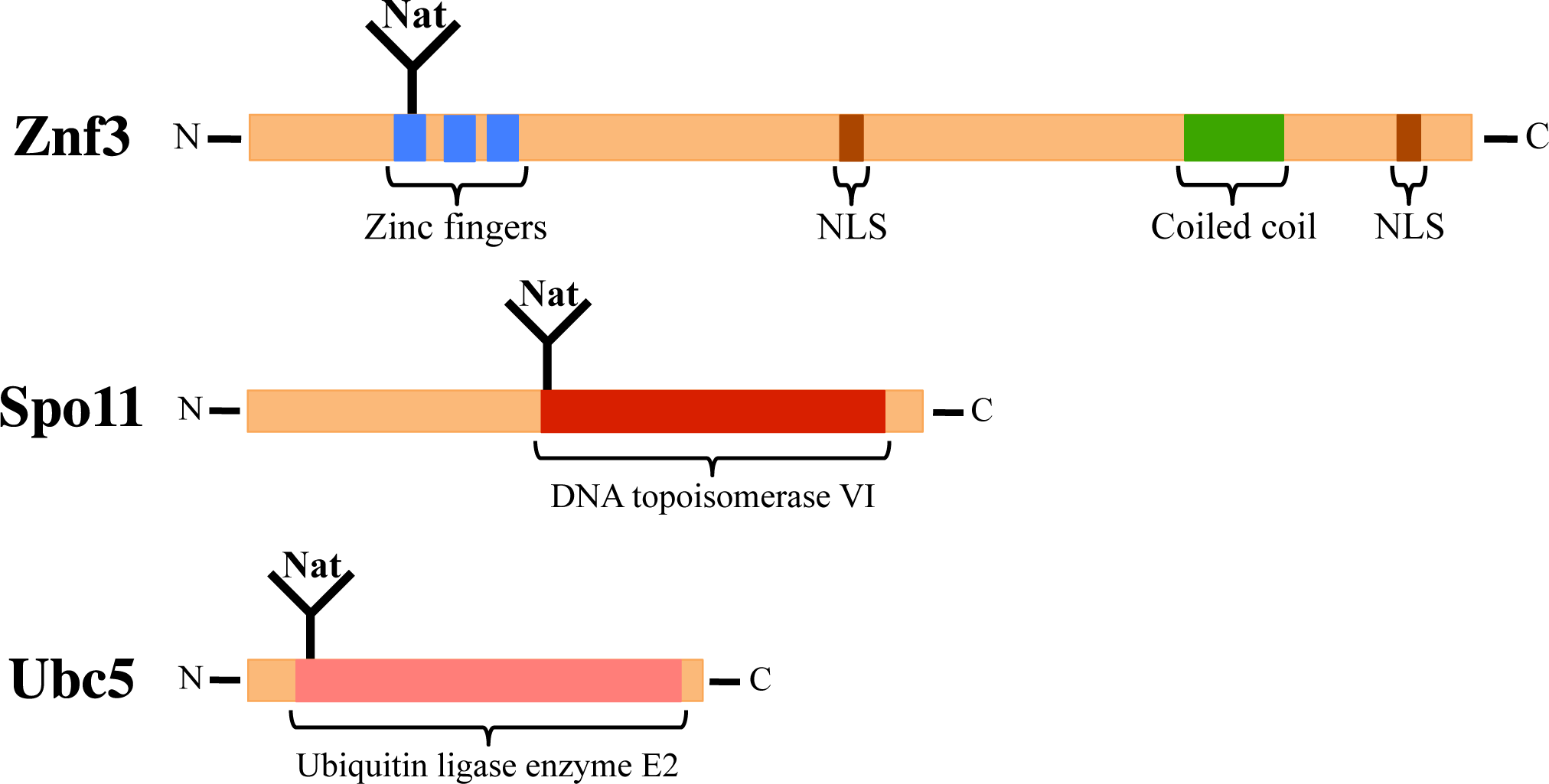

Supplement: Figure S2 — Protein organization of Znf3, Spo11, and Ubc5. Znf3 is a large protein (1,561 amino acids) unique in the Cryptococcus genus. Due to the presence of three zinc finger domains and possible localization to the nucleus based on the presence of predicted nuclear localization signals (NLS), this protein may act as a transcription factor or could be involved in other nucleic acid (DNA or RNA)-regulated processes or trafficking. A coiled coil region could mediate interactions with an unknown co-factor or itself. Spo11 has a conserved DNA topoisomerase VI domain that mediates its role in generating DNA DSBs during meiosis. Ubc5 has a conserved ubiquitin-conjugating E2 enzyme domain that spans the entire protein. In the insertion mutants, the genes have been disrupted at the designated sites by the NAT drug resistance marker. Motif predictions were based on the genome of JEC21α and the motif scan tool PROSITE. Protein localization sites were identified using the WoLF PSORT program. (TIF) [file pgen.1003688.s002.tif]

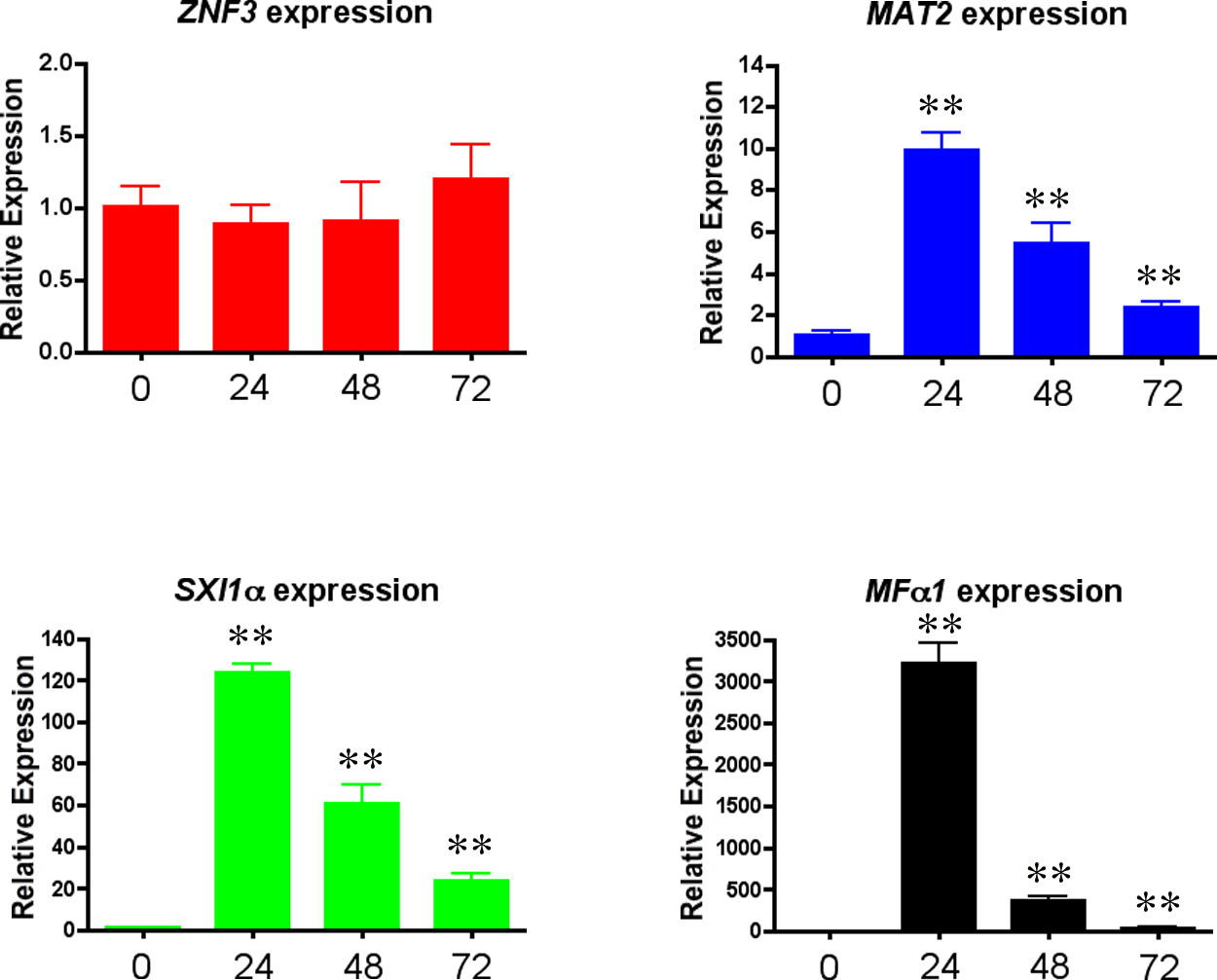

Supplement: Figure S3 — Expression profiles of ZNF3 , MAT2 , SXI1 α and MF α 1 during bisexual reproduction. WT α and a cells were mixed in equal numbers, co-cultured on V8 medium, and incubated in the dark for 0 (vegetative growth), 24, 48, or 72 hrs. The cells were harvested and RNA was isolated from both yeast cells and hyphae. RT-PCR showed that ZNF3 expression during bisexual reproduction remained similar to vegetative growth. MAT2, SXI1α, and MFα1 expression increased significantly at 24 hrs. The error bars represent the standard deviations from the mean for the three biological replicates. (TIF) [file pgen.1003688.s003.tif]

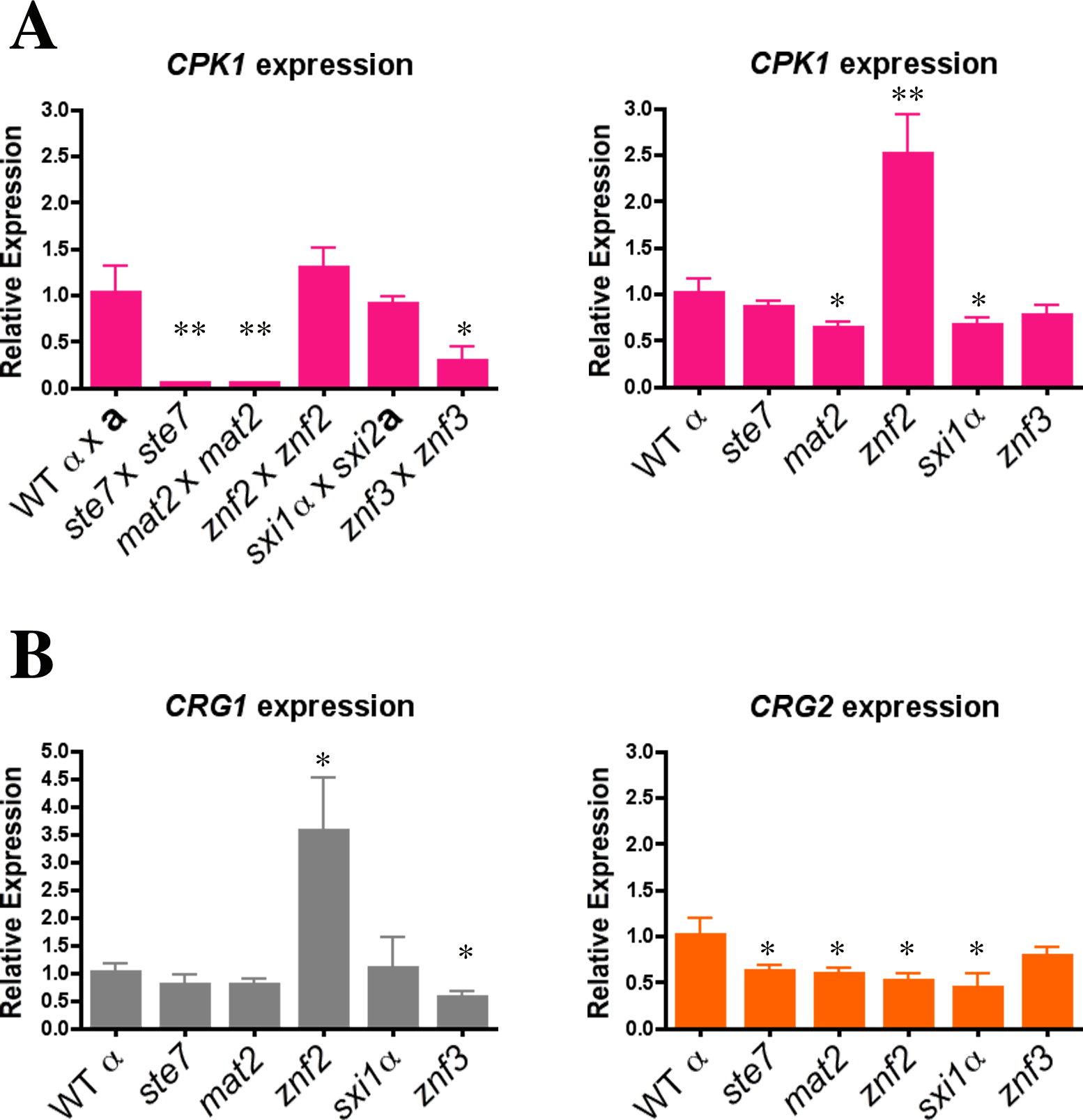

Supplement: Figure S4 — Expression profile of genes encoding Cpk1, Crg1, and Crg2. Cells were incubated for 24 hours on V8 medium for bisexual and 48 hours for unisexual reproduction. Yeast and hyphal cells were harvested and RNA was isolated. Expression was measured by RT-PCR in wild type, ste7Δ, mat2Δ, znf2Δ, sxi1αΔ, and znf3Δ mutants. (A) The MAP kinase CPK1 transcriptional profile is similar to MAT2, and its expression is possibly regulated by the pheromone signaling cascade. (B) Expression of the negative regulator CRG1 was significantly increased in the znf2Δ mutant. The elevated levels of CRG1 may contribute to the severe filamentation defect of znf2Δ mutants during unisexual reproduction. CRG2 expression in pheromone response mutants was similar to wild type (* indicates P<0.05 and ** indicates P<0.005 compared to the WT). The error bars represent the standard deviations from the mean for the three biological replicates. (TIF) [file pgen.1003688.s004.tif]

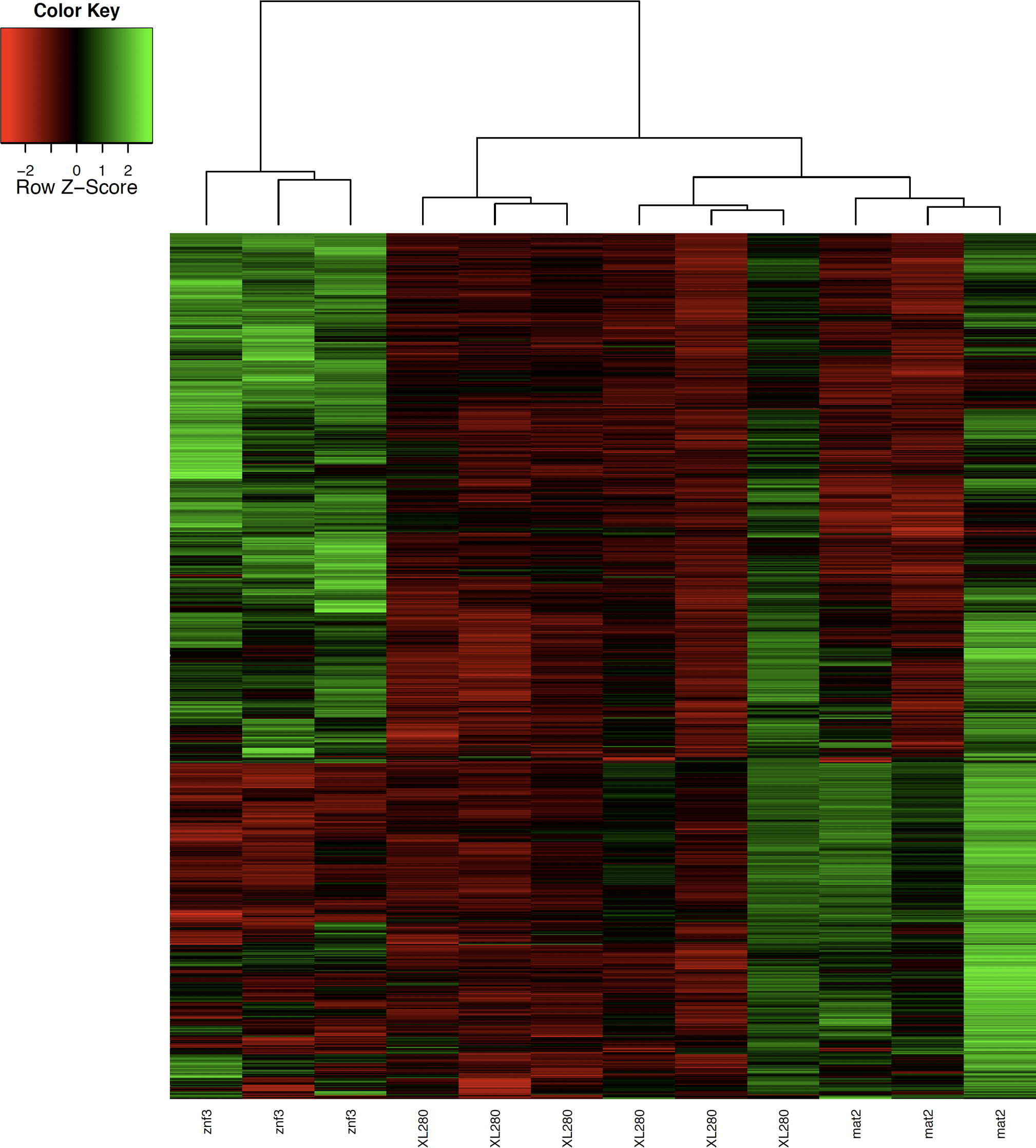

Supplement: Figure S5 — Microarray analysis of gene expression in znf3 Δ, mat2 Δ and XL280 during unisexual reproduction. Microarray data was obtained from three independent experiments for each mutant and the wild type during unisexual reproduction. Hierarchical cluster analysis is presented in the heatmap as z-score normalized log2 expression values. The columns represent each microarray experiment whereas the rows represent the genes filtered by statistical significance (p<0.05). Green indicates an increase in expression level, red indicates a decrease, and black indicates that the expression level did not change between the different isolates. (TIF) [file pgen.1003688.s005.tif]

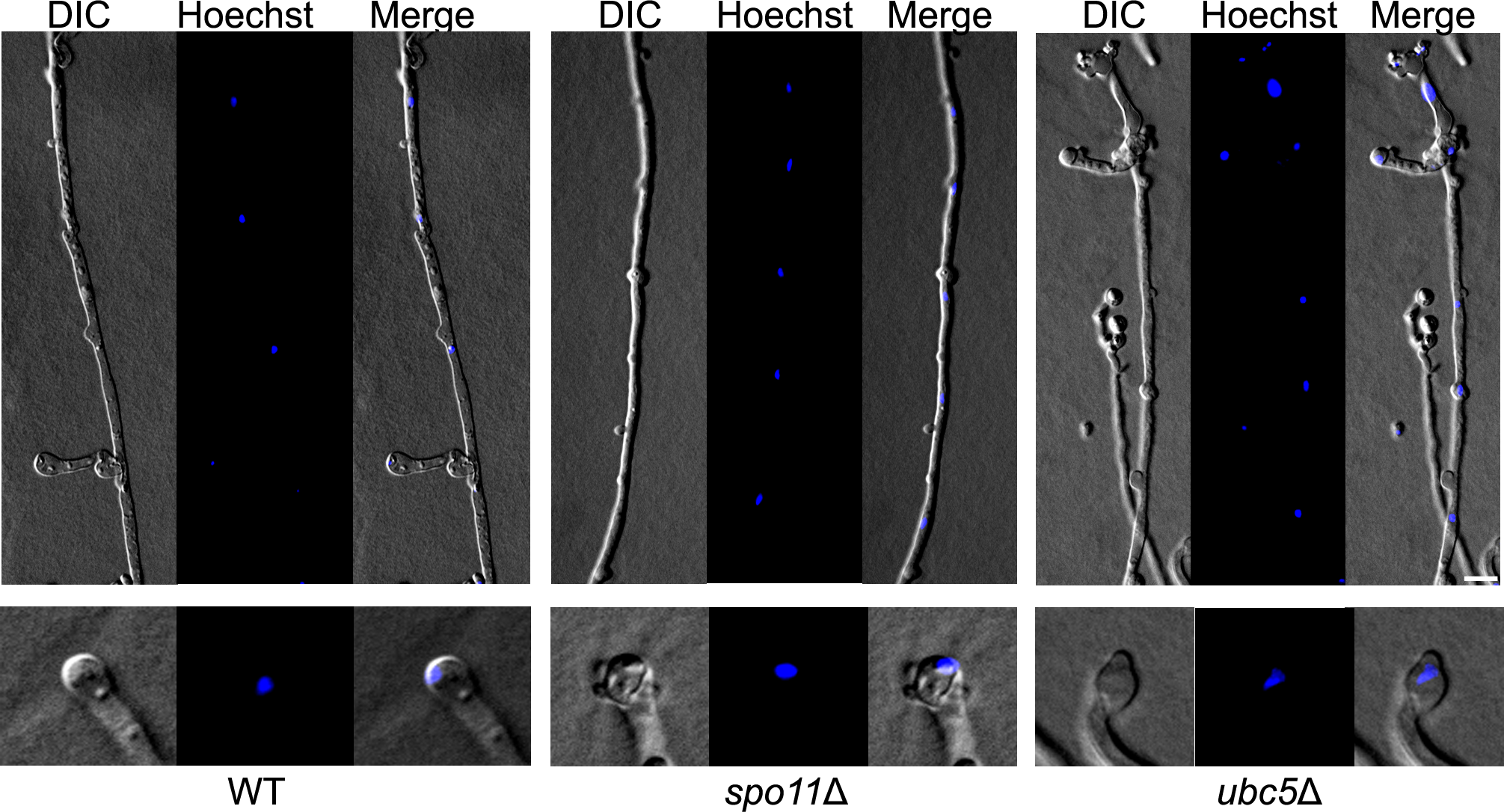

Supplement: Figure S6 — Localization of nuclei during unisexual reproduction. Wild type, spo11Δ, and ubc5Δ mutants were incubated on MS medium in the dark at room temperature for 10 days. Small patches of agar were excised and stained with Hoechst dye. Nuclear positioning was visualized with fluorescent microscopy. In wild type unisexual reproduction hyphae are monokaryotic with distinct nuclei in each hyphal compartment. In spo11Δ and ubc5Δ mutants, the unisexual hyphae are similar to wild type. The kar7Δ karyogamy mutant impairs hyphal growth by preventing early nuclear diploidization, and also leads to a sporulation defect by blocking late nuclear diploidization, leading to paired unfused nuclei in the basidium [37]. The spo11Δ, and ubc5Δ mutants have only one nucleus in the basidium similar to the wild type and no hyphal growth impairment, consistent with no observed defects in karyogamy. The scale bar represents 10 µm. (TIF) [file pgen.1003688.s006.tif]

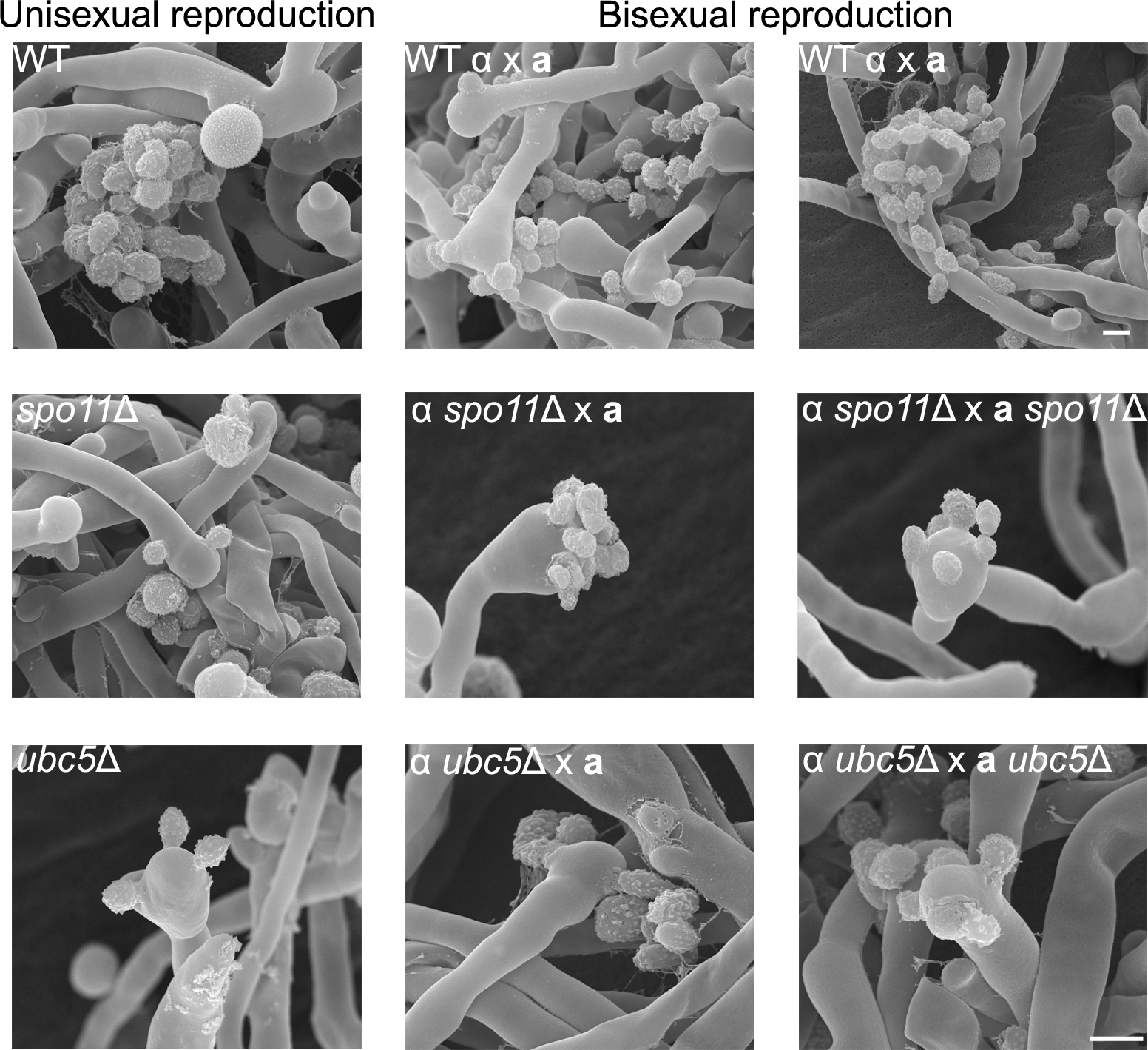

Supplement: Figure S7 — Scanning electron microscopic analysis of sporulation defects. The upper panel shows wild type bisexual and unisexual reproduction leading to hyphae with basidia decorated with long spore chains that formed after 14 days incubation on MS media. The middle and lower panels depict sporulation defects during bisexual and unisexual reproduction of spo11Δ and ubc5Δ mutants, respectively. The scale bars represent 1 µm. (TIF) [file pgen.1003688.s007.tif]

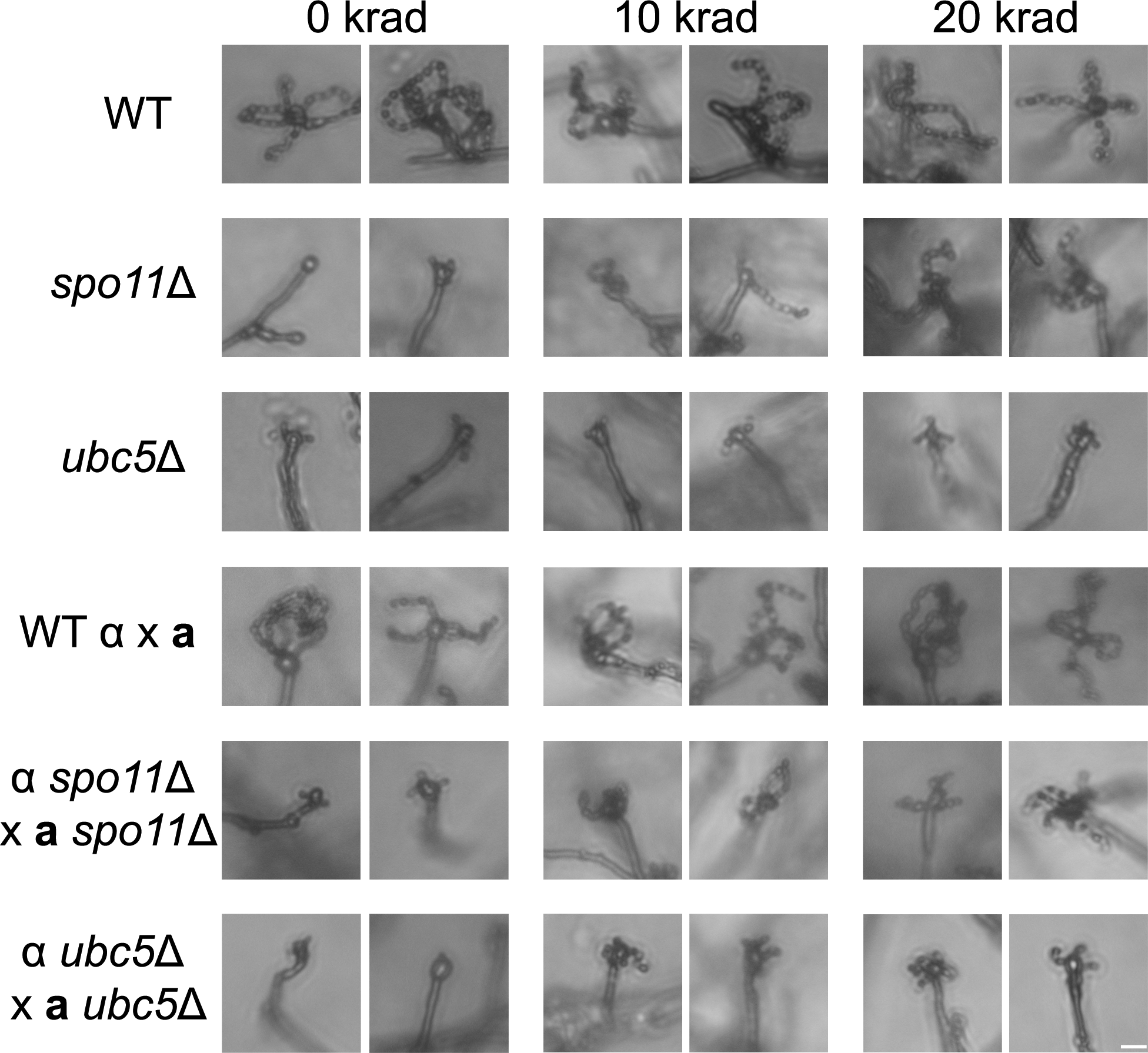

Supplement: Figure S8 — X-irradiation partially suppresses the sporulation defect of spo11 mutants. Bisexual and unisexual reproduction cultures were incubated for 7 days on V8 medium, irradiated with the designated dose, and incubated 2 additional days in the dark at room temperature. The first row depicts the sporulation defect of spo11Δ and ubc5Δ mutants of unirradiated cultures. The second and third rows show that X-irradiation partially restored spore production in the spo11Δ mutant, while sporulation of the irradiated ubc5Δ mutant was similar to the unirradiated samples. The scale bar represents 10 µm. (TIF) [file pgen.1003688.s008.tif]
