# Supplementary material for: Genetic Circuits that Govern Bisexual and Unisexual Reproduction in Cryptococcus neoformans
Source: PLoS Genet. 2013 Aug 15;9(8):e1003688. doi: 10.1371/journal.pgen.1003688 (PMC3744442; doi:10.1371/journal.pgen.1003688)
Supplement: Table S3 — Strains and plasmids used in this study. (DOC) [file pgen.1003688.s011.doc]

**Table S3.** Strains and plasmids used in this study

| **Strain Name** | **Genotype** | **Background** | **Sources and comments** |
| --- | --- | --- | --- |
| XL280α | *MAT*α |  | ~81% genome identity with JEC21α [36] |
| JEC21α | *MAT*α |  | [9] |
| JEC20**a** | *MAT***a** |  | congenic with JEC21 [9] |
| MF01 | *MAT*α *znf3*Δ::*NAT* | XL280α | This study |
| MF38 | *MAT*α *znf3*Δ::*NAT* | XL280α | This study |
| MF39 | *MAT*α *znf3*Δ::*NAT* | JEC21α | This study |
| MF40 | *MAT***a** *znf3*Δ::*NEO* | JEC20**a** | This study |
| MF41 | *MAT***a** *znf3*Δ::*NEO* | JEC20**a** | This study |
| MF29 | *MAT*α *ste7*Δ::*NAT* P*_GPD1_*-*ZNF3* | XL280α | XL946 [26] transformed with pMF1 |
| MF34 | *MAT*α P*_GPD1_*-*ZNF3* | XL280α | This study |
| MF35 | *MAT*α *mat2*Δ::*NAT* P*_GPD1_*-*ZNF3* | XL280α | XL942 [26] transformed with pMF1 |
| MF54 | *MAT*α *znf3*Δ::*NAT* P*_GPD1_*- *MAT2* | XL280α | MF01 transformed with pDX64 (Lin and Heitman, unpublished data) |
| XL143 | *MAT*α/ *MAT*α | JEC21α | [18] |
| XL561 | *MAT*α *NAT* | XL280α | (Lin and Heitman, unpublished data) |
| XL491 | *MAT***a** *NEO* | JEC20**a** | (Lin and Heitman, unpublished data) |
| XL574 | *MAT*α *znf2*Δ::*NAT* | XL280α | [26] |
| XL867 | *MAT***a** *znf2*Δ::*NAT* | JEC20**a** | [26] |
| XL942 | *MAT*α *mat2*Δ::*NAT* | XL280α | [26] |
| XL961 | *MAT***a** *mat2*Δ::*NEO* | JEC20**a** | [26] |
| XL946 | *MAT*α *ste7*Δ::*NAT* | XL280α | [26] |
| RDC25-2 | *MAT***a** *ste7*Δ::*ADE2 ade2* | JEC20**a** | [21] |
| XL1108 | *MAT*α *sxi1*αΔ::*NAT* | XL280α | (Lin and Heitman, unpublished data) |
| CHY766 | *MAT***a** *sxi2***a**Δ::*URA5 ura5* | JEC20**a** | [42] |
| MF42 | *MAT*α *spo11*Δ::*NAT* | XL280α | This study |
| MF43 | *MAT*α *spo11*Δ::*NAT* | XL280α | This study |
| MF44 | *MAT*α *spo11*Δ::*NAT* | JEC21α | This study |
| MF45 | *MAT*α *spo11*Δ::*NAT* | JEC21α | This study |
| MF46 | *MAT***a** *spo11*Δ::*NEO* | JEC20**a** | This study |
| MF47 | *MAT***a** *spo11*Δ::*NEO* | JEC20**a** | This study |
| MF56 | *MAT*α *spo11*Δ::*NAT SPO11*-*NEO* | XL280α | Complementation of MF42 |
| MF57 | *MAT*α *spo11*Δ::*NAT SPO11*-*NEO* | JEC21α | Complementation of MF44 |
| MF58 | *MAT***a** *spo11*Δ::*NEO SPO11*-*NAT* | JEC20**a** | Complementation of MF46 |
| MF48 | *MAT*α *ubc5*Δ::*NEO* | XL280α | This study |
| MF49 | *MAT*α *ubc5*Δ::*NEO* | XL280α | This study |
| MF50 | *MAT*α *ubc5*Δ::*NAT* | JEC21α | This study |
| MF51 | *MAT*α *ubc5*Δ::*NAT* | JEC21α | This study |
| MF52 | *MAT***a** *ubc5*Δ::*NEO* | JEC20**a** | This study |
| MF53 | *MAT***a** *ubc5*Δ::*NEO* | JEC20**a** | This study |
| MF59 | *MAT*α *ubc5*Δ::*NEO UBC5*-*NAT* | XL280α | Complementation of MF48 |
| MF60 | *MAT*α *ubc5*Δ::*NAT UBC5*-*NEO* | JEC21α | Complementation of MF50 |
| MF61 | *MAT***a** *ubc5*Δ::*NEO UBC5*-*NAT* | JEC20**a** | Complementation of MF53 |
| MF121 | *MAT*α *znf3*Δ::*NEO ura5* | XL280α | This study |
| MF165 | *MAT*α *znf3*Δ::*NAT* P*_GPD1_*-*ZNF3* | XL280α | MF01 transformed with pMF1 |
| MF166 | *MAT*α *znf3*Δ::*NAT* P*_GPD1_*-*ZNF3* | XL280α | MF38 transformed with pMF1 |
| pXL1 | P*_GPD1_* *NEO* *AMP* |  | (Lin and Heitman, unpublished data) |
| pJAF12 | *NEO* *AMP* |  | Used for complementation [14] |
| pJAF13 | *NAT* *AMP* |  | Used for complementation [14] |
| pDX64 | P*_GPD1_*-*MAT2* *NEO* *AMP* | pXL1 | (Lin and Heitman, unpublished data) |
| pMF1 | P*_GPD1_*-*ZNF3* *NEO* *AMP* | pXL1 | This study |
| pMF44 | *UBC5 NEO* *AMP* | pJAF12 | This study |
| pMF46 | *UBC5 NAT* *AMP* | pJAF13 | This study |
| pMF52 | *SPO11 NEO AMP* | pJAF12 | This study |
| pMF54 | *SPO11 NAT AMP* | pJAF13 | This study |
