# Supplementary material for: Genetic Circuits that Govern Bisexual and Unisexual Reproduction in Cryptococcus neoformans
Source: PLoS Genet. 2013 Aug 15;9(8):e1003688. doi: 10.1371/journal.pgen.1003688 (PMC3744442; doi:10.1371/journal.pgen.1003688)
Supplement: Table S4 — Primers used in this study. (DOC) [file pgen.1003688.s012.doc]

**Table S4.** Primers used in this study

| **Primer name** | **Sequence (5’ to 3’)** | **Description** |
| --- | --- | --- |
| M13F | GTAAAACGACGGCCAG |  |
| M13R | CAGGAAACAGCTATGAC |  |
| AI076 | AACAGTTGCGCAGCCTGAATG | Primer for inverse PCR [29] |
| AI077 | AGAGGCGGTTTGCGTATTGG | Primer for inverse PCR [29] |
| JOHE8744 | AGCAAAGCGAAAAGCT | *BWC1* screening |
| JOHE8745 | ATACGCCTACCTACGTGCTAGC | *BWC1* screening |
| JOHE17111 | GGAGGGAAAAGGAACCTTG | *BWC2* screening |
| JOHE17111 | CTAACTGGGCTGTTTCAATC | *BWC2* screening |
| JOHE13684 | CGCAGGGCGAAACTTTGTG | *ZNF2* screening |
| JOHE13683 | CCTCATCTCTCGCTCAGCCTC | *ZNF2* screening |
| JOHE14082 | CATCCCTTATCCTCACTGCC | *MAT2* screening |
| JOHE14088 | GCTTTCCCTCCGCTTCCTG | *MAT2* screening |
| JOHE14068 | GCGCTGGGCAAGATGCAAG | *STE7* screening |
| JOHE14074 | GGCCGACCATGGATTGAGC | *STE7* screening |
| JOHE20489 | AAAGCTTTTTAATCGTCAGTCT | *CPK1* screening |
| JOHE20494 | TACATCCACCACCCGATCCTTA | *CPK1* screening |
| JOHE20806 | GTCACTATGTGTTACAGAGCTG | *CRG1* screening |
| JOHE20809 | ACGAACTCTTGACATTGTCGTCAAC | *CRG1* screening |
| JOHE15244 | GCCTTCTTGCCACTGTCG | *SPO11* disruption |
| JOHE15245 | CTGGCCGTCGTTTTACCAACCCAGAAGGACAACCG | *SPO11* disruption |
| JOHE15246 | GTCATAGCTGTTTCCTGCACATCTATGGCTAGCGATAGTG | *SPO11* disruption |
| JOHE15247 | GCCCACAAAGTCTTTCGAC | *SPO11* disruption |
| JOHE15248 | ATGCTTCGTACAATGCCTTC | *spo11* screening |
| JOHE15250 | CGTCAGGAAGAGGCTGCC | *spo11* screening |
| JOHE20738 | CTGATTTCGCCTTCTTCCTTGT | *ZNF3* disruption |
| JOHE20745 | AGCATCTAGGACTAAAAGTCAGC | *ZNF3* disruption |
| JOHE20800 | CTGGCCGTCGTTTTACGGGGTTAGTAGCTGGCTGTTT | *ZNF3* disruption |
| JOHE20801 | GTCATAGCTGTTTCCTGGTGGGGACAGCAATTCATATAAG | *ZNF3* disruption |
| JOHE20739 | AGCTGCATTTGATGGATGGAAC | *znf3* screening |
| JOHE20740 | TGAGCTTCCTATCTTACGTCTC | *znf3* screening |
| JOHE20741 | TTGCTAGAAGTCAAGTCGTCCT | *znf3* screening |
| JOHE20742 | AAGACGTCGACTTTGAGCTTCCT | *znf3* screening |
| JOHE20743 | CTCACTGCTGAAGGGTAGATCTTA | *znf3* screening |
| JOHE20744 | AGGAGTTCAAGCTTCAATGGTG | *znf3* screening |
| JOHE21470 | ATTATGGCCGGCCATGCTCACAGCCTTCTTCGGCTACGTTA | *ZNF3* overexpression |
| JOHE21471 | ACACGCCTTAATTAATCACCAGCCACCAGTGTCTCCGTCT | *ZNF3* overexpression |
| JOHE24424 | TGAAAACAACTACGACGGTCGGTACATTGAGCC | *ZNF3* RT-PCR |
| JOHE24425 | ATCCATCCTGTTGGAGAGCAGCTG | *ZNF3* RT-PCR |
| JOHE24426 | AGGAAGCTCGAGCATCGAAAGCTGTAT | *MAT2* RT-PCR |
| JOHE24427 | TGAGCTGCAGTAGCTCGTAAATCTGAC | *MAT2* RT-PCR |
| JOHE24428 | ACCCTTTCCAACCCCTTGTCAACG | *ZNF2* RT-PCR |
| JOHE24429 | AAGGACGTTTCCCAGTGTGAATACGCC | *ZNF2* RT-PCR |
| JOHE24430 | GTCACTTTTGCTAGATGAAGGGCAAAGTCG | *SXI1*α RT-PCR |
| JOHE24431 | GATCACCACCACACCACACGTTCA | *SXI1*α RT-PCR |
| JOHE24434 | CTACCGACCAGCAACCAACCATCGCTAC | *MF*α*1* RT-PCR |
| JOHE24435 | GTCAATACCATCTAAACAAGTCCCATACGCTTC | *MF*α*1* RT-PCR |
| JOHE24436 | CAGGTTCAACGTCGGCAACAACTA | *CPK1* RT-PCR |
| JOHE24437 | TCAAGTCGCGATGGATGATTTCAGCAG | *CPK1* RT-PCR |
| JOHE26873 | GCCGCCGTTAATTGGATGTTGGAT | *CRG1* RT-PCR |
| JOHE26874 | AGTGATGTACGGCGTTGTACGGAA | *CRG1* RT-PCR |
| JOHE26875 | TGCGAGCTAAAGCCTTTGCCAATC | *CRG2* RT-PCR |
| JOHE26876 | AAAGTACCGACGCTCCGACAATGA | *CRG2* RT-PCR |
| JOHE37497 | TGCCGCAATTACGACCACGT | *UBC5* disruption |
| JOHE37498 | CTGGCCGTCGTTTTACCAGGCGTTGAATTAAACGGT | *UBC5* disruption |
| JOHE37499 | GTCATAGCTGTTTCCTGAAGTCCTTGTAACAATATCTT | *UBC5* disruption |
| JOHE37500 | TATCGACTCTCTCGGAGAGGAGTT | *UBC5* disruption |
| JOHE37501 | ACCAATAGGCACTCCAAAGAGCG | *ubc5* screening |
| JOHE37502 | TTCCGCCTGCATCATTCTAACGT | *ubc5* screening |
| JOHE37665 | ATTACTCGAGCTTCTTGAAGGCTTTCGTAG A | *SPO11* complementation |
| JOHE37666 | ATTACTCGAGCCAAGGCCAAACAAAGTGTTT | *SPO11* complementation |
| JOHE37667 | TATGTTCTAGAGAACGCACGACTTTCAGATGT | *UBC5* complementation |
| JOHE37668 | TATGTTCTAGACATGAGAACTCCAGGGCATAA | *UBC5* complementation |
