# Supplementary material for: Genetic Circuits that Govern Bisexual and Unisexual Reproduction in Cryptococcus neoformans
Source: PLoS Genet. 2013 Aug 15;9(8):e1003688. doi: 10.1371/journal.pgen.1003688 (PMC3744442; doi:10.1371/journal.pgen.1003688)
Supplement: Text S1 — Genomic and protein organization of Znf3, Spo11, and Ubc5. (DOC) [file pgen.1003688.s013.doc]

**Text S1**

**Genomic and protein organization of Znf3, Spo11, and Ubc5**

The genomic organization of the *ZNF3* gene is conserved in the A and D serotypes and in *C. gattii* molecular type VGI. The *ZNF3* gene is located on chromosome 11 in serotype D. Interestingly, the gene is completely deleted in the *C. gattii* VGII molecular type, including the Pacific Northwest outbreak VGIIa major strain R265, while the organization of the flanking genes remains conserved between *C. gattii* VGI and VGII [1,2]. Whole genome sequencing of XL280α revealed that the *ZNF3* sequence originated from the B3501α parent and contains an additional 138 nucleotide region compared to JEC21α. Surprisingly, this region is also absent from *C. gattii* VGI but it is present in the serotype A strain H99α. The XL280α *ZNF3* gene encodes a protein with 1,561 amino acids with three N-terminal zinc finger C2H2 domains, two nuclear localization signals (NLS), and a C-terminal coiled-coil region (Figure S1). The first zinc finger is encoded by the additional sequence present in B3501α and H99α, but it is missing in JEC21α and *C. gattii*. Because zinc finger domains are common features of transcription factors and due to the presence of two NLS signals, we hypothesized that Znf3 localizes in the nucleus and could play a role as a transcription factor. On the other hand, C2H2 zinc finger domains have also been observed to recognize both DNA and RNA, implicating the protein in nucleic acid trafficking or other roles involving RNA binding such as mRNA stabilization or activation or repression of translation [3]. A BLASTP search did not yield significant similarity with proteins outside the *Cryptococcus* genus, indicating a possible species-specific role for this candidate novel regulator.

The *SPO11* gene is conserved throughout the *Cryptococcus* species. The *SPO11* gene lies on chromosome 8 and encodes a 558 amino acid protein with a conserved DNA topoisomerase VI domain (Figure S1). A BLASTP search revealed significant similarity with previously identified proteins in *Coprinopsis cinerea* (38% identity, 55% similarity) [4], *Homo sapiens* (34% identity, 54% similarity) [5], *Schizosaccharomyces pombe* (35% identity, 52% similarity) [6], and *Saccharomyces cerevisiae* (25% identity, 45% similarity) [7]. Most of the similarity occurs in the C-terminal domain of the protein. Spo11 is a meiosis-specific endonuclease that catalyzes the formation of DNA double-strand breaks required for meiotic recombination. Deletion of the gene in *S. cerevisiae* decreases the efficiency of recombination and results in sporulation defects, similar to the phenotype of a *spo11* insertion mutant in *C. neoformans* [7].

The *UBC5* gene is located on chromosome 4 and is unlinked to the mating type locus, which is also located on chromosome 4. The gene encodes a small protein of 135 amino acids with a ubiquitin-conjugating enzyme E2 catalytic (UBC) domain that spans 128 amino acids (Figure S1). The protein is highly conserved among *Cryptococcus* species (94 – 96% identity) and in the fungal kingdom. The predicted protein shows high homology with proteins from *Ustilago maydis* (54% identity, 65% similarity), *Schizosaccharomyces pombe* (50% identity, 68% similarity), *Aspergillus fumigatus* (48% identity, 68% similarity), and *Saccharomyces cerevisiae* (25% identity, 45% similarity). Ubiquitin-conjugating E2 enzymes are responsible for attaching ubiquitin to a lysine residue of a protein targeted for degradation. In *S. cerevisiae* Ubc5, together with Ubc4, plays a role in the degradation of short-lived proteins and deletion of the gene causes growth and sporulation defects, although the molecular basis of this phenotype is unknown [8,9].

**References**

1. D'Souza CA, Kronstad JW, Taylor G, Warren R, Yuen M, et al. (2011) Genome variation in *Cryptococcus gattii*, an emerging pathogen of immunocompetent hosts. mBio 2: e00342-00310.

2. Gillece JD, Schupp JM, Balajee SA, Harris J, Pearson T, et al. (2011) Whole genome sequence analysis of *Cryptococcus gattii* from the Pacific Northwest reveals unexpected diversity. PLoS One 6: e28550.

3. Hall TM (2005) Multiple modes of RNA recognition by zinc finger proteins. Curr Opin Struct Biol 15: 367-373.

4. Celerin M, Merino ST, Stone JE, Menzie AM, Zolan ME (2000) Multiple roles of Spo11 in meiotic chromosome behavior. EMBO J 19: 2739-2750.

5. Romanienko PJ, Camerini-Otero RD (1999) Cloning, characterization, and localization of mouse and human *SPO11*. Genomics 61: 156-169.

6. Wood V, Gwilliam R, Rajandream MA, Lyne M, Lyne R, et al. (2002) The genome sequence of *Schizosaccharomyces pombe*. Nature 415: 871-880.

7. Klapholz S, Waddell CS, Esposito RE (1985) The role of the *SPO11* gene in meiotic recombination in yeast. Genetics 110: 187-216.

8. Seufert W, Jentsch S (1990) Ubiquitin-conjugating enzymes *UBC4* and *UBC5* mediate selective degradation of short-lived and abnormal proteins. EMBO J 9: 543-550.

9. Hochstrasser M (1996) Ubiquitin-dependent protein degradation. Annu Rev Genet 30: 405-439.
